# Supercapacitive CO_2_ Capture through a Scalable, Aqueous, Neutral Polymer‐Based Electrolyte

**DOI:** 10.1002/cssc.202501259

**Published:** 2025-09-22

**Authors:** Daniel García‐Giménez, Marta Santos‐Rodríguez, Antoniou Maria‐Anna, Mohammad Sanan‐Ali, Miguel A. López‐Manchado, Javier Carretero‐González

**Affiliations:** ^1^ Institute of Polymer Science and Technology ICTP CSIC 28006 Madrid Spain

**Keywords:** capacitors, CO_2_ capture, electrochemical cells, energy storage, polymer electrolyte

## Abstract

Aqueous electrolytes offer a safer, more cost‐effective, and scalable solution for electrochemical CO_2_ capture. Herein, electrolytes that combine harmless polyethylene glycol with slightly salty water are developed, which enables the capture of CO_2_ in a supercapacitor cell of up to 79 mmol of CO_2_ per kilogram of electrode material while operating at a current density of 30 mA g^−1^, a voltage of 2.5 V, at 40 °C, and under neutral pH conditions. Interestingly, applying negative charge protocols up to −2.5 V increases the amount of CO_2_ adsorbed per kilogram of electrode to 356 mmol. This is attributed to both the ejection of preadsorbed bicarbonate species from the pores and their slow migration kinetics near the electrode exposed to CO_2_ gas toward the positively charged counter electrode. This finding indicates that the CO_2_ capture and release mechanism exhibits no dependence on the direction of the charge. These polymer electrolytes lead to a threefold increase in the gravimetric energy stored compared to similar devices operating at ≈1 V that utilize microporous carbon electrode materials. The system also reveals excellent stability and corrosion resistance under long‐term cycling at high voltages. This advanced technology marks a major advancement in sustainable carbon capture solutions.

## Graphical abstract

Slowly but surely. Safe, pH‐neutral, and scalable water‐based polymer electrolyte allows for the capture of CO_2_ within a supercapacitor cell. By applying negative charge protocols of up to −2.5 V, it achieves the capture of up to 356 mmol of CO_2_ per kilogram of electrode material. This process likely occurs due to the slow migration of bicarbonate species toward the counter electrode .

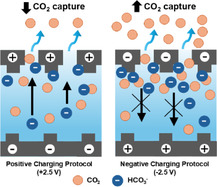

## 1 Introduction

The global earth temperature increase during the last century is highly correlated with the carbon dioxide (CO_2_) concentration in the atmosphere, which was provoked mainly by human activities such as the burning of fossil fuels.^[^ ^1^, ^2^ ^]^ Global CO_2_ emissions have currently reached a historical high of more than 35 GtCO_2_ and a global average atmospheric carbon dioxide beyond 420 ppm.^[^ ^3^ ^]^ These CO_2_ levels today are higher than at any point in at least the past 800,000 years.^[^ ^4^ ^]^ Therefore, carbon capture is essential for mitigating CO_2_ emissions and stopping global warming.

Wet chemical scrubbing using amines to remove CO_2_ from a mixture of gases is the most employed carbon capture technology at the million‐ton scale.^[^ ^5^ ^]^ However, this technology still presents some cost and environmental issues, such as: i) the energy required for the regeneration of the solvents and the large quantities of water needed; ii) the amines can escape into the environment and could be toxic to life, so by‐products must be treated to avoid environmental disasters.^[^ ^6^, ^7^ ^]^ For this reason, sustainable CO_2_ capture alternatives are being sought.^[^ ^8^ ^]^ Among them, the supercapacitive swing adsorption (SSA) by using carbon porous electrode materials is a promising strategy.^[^ ^9^ ^]^ In the SSA, the regeneration of the sorbent is performed by changing the voltage difference in the supercapacitor cell instead of applying temperature to release the CO_2_ captured. According to the Biejrrun diagram,^[^ ^10^ ^]^ within this pH range, CO_2_ is in equilibrium with negatively charged bicarbonate ions. Bicarbonate ions are released from the electric double layer at the surface of the negatively charged gas‐exposed porous carbon electrode during the discharge process, being presumably the driving force to dissolve more CO_2_ into aqueous polymer electrolytes, as supported by recent studies on the operating mechanisms.^[^ ^11^, ^12^ ^]^

Over the past decade, a comprehensive set of energy and adsorptive metrics has been analyzed to evaluate the properties and performance of SSA of CO_2_ devices.^[^ ^13^ ^]^ Key factors examined include charging protocols^[^ ^13^, ^14^ ^]^ electrochemical voltage value,^[^ ^15^ ^]^ electrolyte concentrations,^[^ ^16^ ^]^ and compositions^[^ ^17^ ^]^ The role of carbon electrodes has also been investigated.^[^ ^18^, ^19^ ^]^ In summary, we can confirm that higher adsorption capacities often come at the cost of reduced coulombic and energy efficiency values.^[^ ^20^ ^]^ The adsorption capacity is a crucial factor in the techno‐economic analysis of the SSA of CO_2_, yet significant cost reductions can also be attained by improving the energy storage of the supercapacitor cell. This is important because electricity costs directly influence the overall operational energy cost per ton of CO_2_ captured. Therefore, further investigation is still required.

Inspired by a recent breakthrough in polyethylene glycol (PEG) concentrated electrolytes for battery applications,^[^ ^21^ ^]^ we have developed a series of formulations using aqueous PEG electrolytes. These electrolytes contain a low sodium chloride (NaCl) concentration of 0.2 M and feature various PEG‐to‐water weight percentages, resulting in a neutral or near‐neutral pH. These formulations demonstrate a broad electrochemical stability window of up to 3 V, which can increase the energy stored in the supercapacitor cell up to three times compared to other aqueous electrolyte SSA systems operating at 1 V. The improvement of the electrochemical window is due to a balance between the interactions among water molecules and between water and the polymer. We have drawn this conclusion for each electrolyte formulation based on dynamic scanning calorimetry and ^1^H nuclear magnetic resonance (NMR) analysis. Notably, the presence of CO_2_ also influences this balance, reducing the mobility of polymer chains in aqueous solutions. When the cell containing the aqueous polymer electrolyte was charged from 0 V to 2.5 V, 79 mmol of CO_2_ per kilogram of electrode was captured, and a clear relationship was observed between the increase in pressure and the duration of the voltage hold. It appears that longer exposure to voltage facilitates the transport of bicarbonate ions, which suggests a probable kinetically limited mechanism for the SSA of CO_2_ with the aqueous polymer electrolytes. This contrasts with previous publications, where the increase in the holding time at a certain voltage moves the system toward the equilibrium, dropping the adsorption capacity of CO_2_. Interestingly, applying negative charge protocols (NCPs) up to −2.5 V increased the amount of CO_2_ adsorbed per kilogram of electrode to 356 mmol. This is attributed to both the ejection of pre‐adsorbed bicarbonate species from the pores and their slow migration kinetics near the electrode exposed to CO_2_ gas toward the positively charged counter electrode (CE). Besides, this finding indicates that the CO_2_ capture and release mechanism with the polymer electrolyte behaves similarly to a symmetric system, exhibiting no dependence on the direction of the charge. In addition, corrosion is practically mitigated with the polymer electrolyte even for high voltages and long holding times, which is usually a reason to observe corrosion.^[^ ^19^ ^]^ Under these pH conditions, our aqueous polymer electrolyte meets the criteria for electrochemical stability and salt solubility, being fully compatible with the SSA of CO_2_, while minimizing competing reactions, corrosion, and also supporting our initial hypothesis and making these aqueous‐based polymer electrolytes used in SSA a promising and sustainable alternative to amine scrubbing.

## 2 Results and Discussion

### 2.1 Study of the Physical and Electrochemical Properties of PEG‐H_2_O‐NaCl Mixtures with and without CO_2_

The acid‐base behavior of the polymer electrolyte compositions studied is essential to evaluate their capacity to capture CO_2_, since this is favored in conditions where the acid‐base equilibrium allows the formation of bicarbonate species (HCO_3_^−^), essential intermediates in the adsorption processes.^[^ ^10^ ^]^ In this context, pH values were analyzed in the presence of air and CO_2_ (Figure S5A, Supporting Information), observing a similar trend in both atmospheres: pH value decreases progressively as the water content in the mixture increases. This evolution is justified by the more acidic character of water compared to PEG, which has a pH value close to neutral. Furthermore, when comparing both atmospheres, a further reduction in pH was evident in the presence of CO_2_. This decrease can be attributed to the dissolution of CO_2_ in the solution, which promotes the formation of acid‐base species such as H_2_CO_3_ and, consequently, HCO_3_^−^, shifting the equilibrium toward a more acidic environment in all the compositions evaluated.

In addition, the viscosity of the electrolytes in both atmospheres was also studied (Figure S5B, Supporting Information), showing a direct correlation between the polymer content and the increase in viscosity, independently of the present gas. However, a notable increase in viscosity was detected under a CO_2_ atmosphere with respect to air. This effect has been previously described in aqueous NaCl solutions at high pressure (50 bar) by Islam et al.,^[^ ^22^ ^]^ and could be due to the interactions between the dissolved CO_2_ and water molecules, which originate a denser structural network in the medium, reducing the relative mobility and increasing the overall viscosity. Finally, the ionic conductivity of the electrolyte compositions (Figure S5C, Supporting Information) showed relatively low values, although it increased with water content, as water favors ionic dissociation and enhances the mobility of the ions. Conductivity differences between atmospheres, however, were insignificant, suggesting that, despite the interactions of CO_2_ with the medium, the conductivity is not substantially affected under the working conditions here studied. These results lead us to conclude that adjusting the composition, particularly the water and polymer content, not only modulates the properties of acid‐base balance, viscosity, and ionic conductivity but also may directly influence electrochemical CO_2_ capture when used as an electrolyte material.

#### 2.1.1 Differential Scanning Calorimetry (DSC)

**Figure 1:**
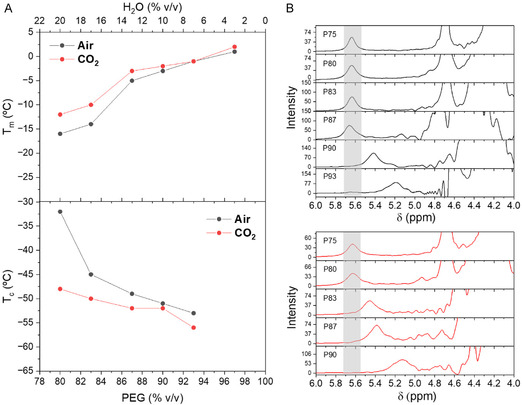
PEG‐H_2_O‐NaCl electrolytes from P75 to P97 with a concentration of NaCl of 0.2M. A) Differential scanning calorimetry experiment. Air atmosphere (black dot‐line‐dot) and CO_2_ atmosphere (red dot‐line‐dot). In the top figure, the melting (*T*_m_) and in the bottom figure, the crystallization temperature (*T*_c_) values are shown, respectively. B) Liquid ^1^H‐NMR experiments using D_2_O as solvent of the different electrolyte mixtures in air (top) and CO_2_ (bottom) atmospheres are shown, respectively.

The molecular dynamics of PEG‐H_2_O‐NaCl solution mixtures with and without CO_2_ were inferred from the thermal properties, such as melting temperature and crystallization temperature, by using the DSC technique (Figure S6). The compositions of the mixtures studied range from 75 v/v% PEG and 25 v/v% H_2_O (P75) to 97 v/v% PEG and 3 v/v% H_2_O (P97). **Figure** **1**A shows the trend evidenced for the crystallization temperature (*T*_c_) and the melting temperature (*T*_m_) values extracted from the second heating ramp in the interval of temperature from −90 °C to 60 °C for all the mixtures. The measurements were carried out under an air (black dots) and CO_2_ (red dots) gas atmosphere. As was expected, the melting temperature values of the mixtures (Figure 1A, top) decreased as the content of water increased, following the same tendency independently of the gas atmosphere. The lowering of the *T*_m_ values indicates that less energy is needed for achieving the phase change from solid to liquid, due to the higher mobility of the polymer chains in the presence of a higher amount of water molecules. Interestingly, when CO_2_ gas was introduced, the melting temperature values were slightly higher for the whole series of mixtures studied under an air atmosphere. This difference seems even more pronounced as the water content increases. The increase in the melting energy is most probably due to a more hindered dynamics of the PEG chains in the presence of CO_2_. In fact, this effect was also observed in the evolution of the *T*_c_ values of the mixtures (Figure 1A, bottom). The *T*_c_ values increased with the water content in the mixtures, regardless of whether an air or a CO_2_ gas atmosphere is present. However, more energy is required for cooling because the polymer mobility is less hindered when CO_2_ is absent. When CO_2_ is introduced, the *T*_c_ values decreased for the whole series in comparison with the gas atmosphere series, indicating less mobility of the PEG chains. This confirms what was also observed during the melting process. In the composition P75, only the glass transition temperature (Tg) is observed; neither the crystallization peak nor the melting peak is present. K. Nishinari and colleagues attributed this to a phenomenon known as perfect supercooling. In this composition, a crystalline structure cannot form during the cooling process, resulting in the maintenance of an amorphous structure.^[^ ^23^ ^]^

#### 2.1.2 Proton (^1^H NMR) Measurements

Information about the dynamics of the polymer electrolytes and their correlation with the amount of water in the presence of CO_2_ can also be inferred from the study of the chemical shift corresponding to the proton in the hydroxyl end group of the PEG chains. This chemical shift is directly related to the electron density around the proton nuclei. In mixtures with a higher content of water, the local environment has a greater electron density, resulting in a displacement toward higher chemical shift values. Consequently, ^1^H‐NMR was conducted on the PEG‐H_2_O‐NaCl series as shown in Figure 1B in both air (top) and CO_2_ (bottom) atmospheres. In both figures, two groups of clear signals can be observed, one group centered at ≈4.25 ppm, which corresponds to the proton resonances in the carbon skeleton of the polymer, and another one centered at 5.5 ppm, corresponding to the proton bonded to the oxygen atom forming the ‐OH group of the polymer chain. In our analysis of the different polymer electrolyte compositions, we did not observe a significant change in the resonances centered at 4.25 ppm. This differs from the findings of Jora M. et al., who studied mixtures of PEG 300 and water.^[^ ^24^ ^]^ In their research, they noted a shift of this signal toward lower values as the polymer content increased. This shift is attributed to a helical conformation that reduces the interaction between water molecules and the polymer chains.^[^ ^24^ ^]^ We did not observe this effect in our study, likely because we focused on compositions with significantly higher polymer content, and this polymer conformation may not have formed. However, in the case of the signal centered at 5.5 ppm, for those compositions exhibiting higher polymer content, shifted toward lower displacement values. This is because the lower the amount of water, the lower the probability of hydrogen bond formation between the proton in the hydroxyl end group of PEG and the oxygen atom of the water molecule; subsequently, there is less de‐shielding and the displacement value of this signal decreases. The behavior in both atmospheres is similar, but in the presence of CO_2_, the resonance shifted in those polymer electrolyte compositions with higher water content, such as P83. This is most likely due to the bicarbonate anions enhancing to a greater extent than in the absence of CO_2_, their interaction with the end groups of the polymer through hydrogen bonds. These results support the observations from the DSC measurements and confirm the impact of CO_2_ on the polymer electrolyte dynamics.

#### 2.1.3 Electrochemical Measurements

**Figure 2:**
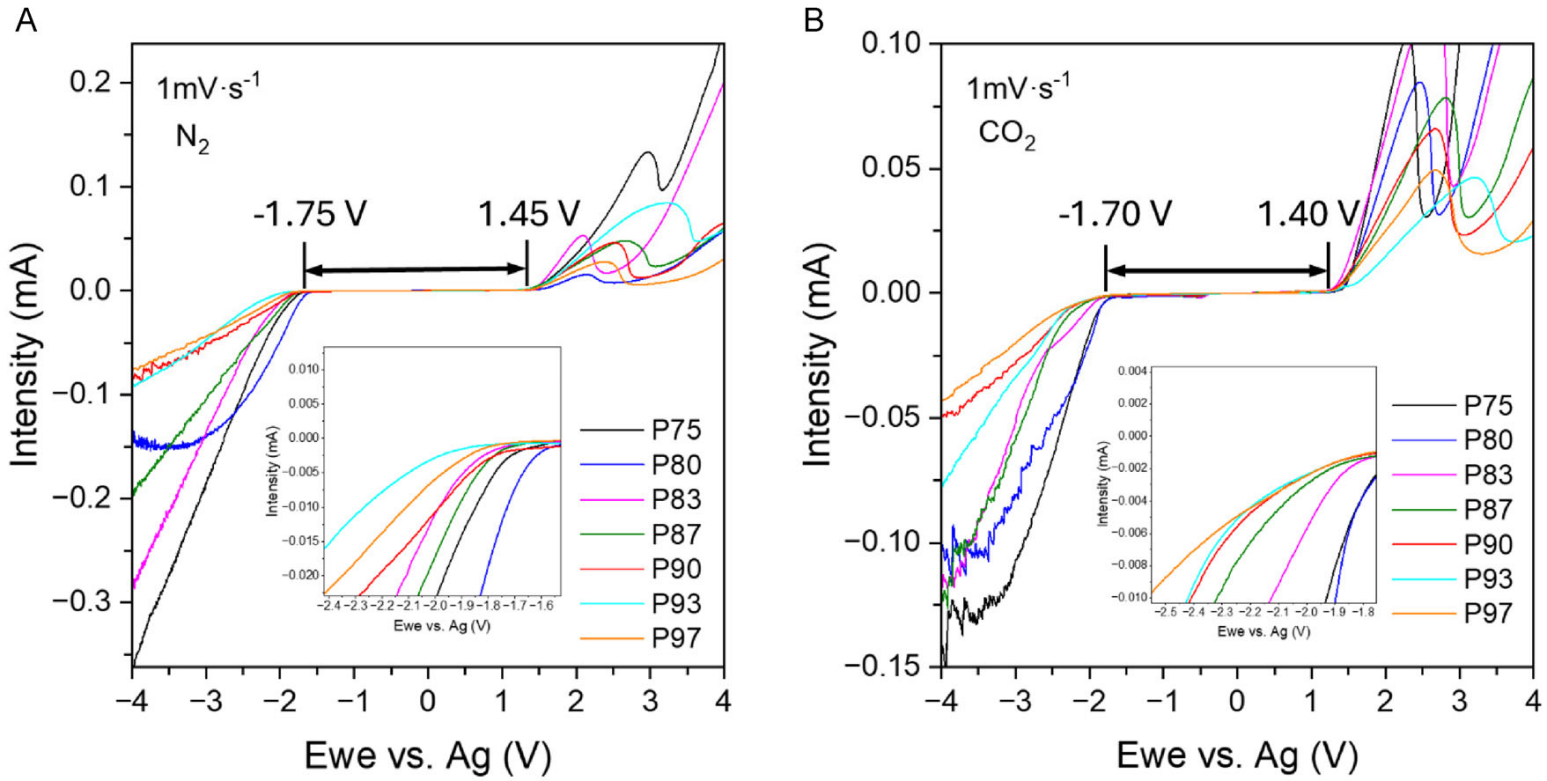
Electrochemical stability window in a three‐electrodes cell using LSV technique for PEG‐H_2_O‐NaCl electrolytes in A) N_2_ atmosphere; B) CO_2_ atmosphere; Scan rate: 1 mV·s^−1^, Potential window studied: 5 V to −5 V versus Ag. Insets: a zoomed view of the negative potential region to better observe the electrochemical behavior at cathodic potentials.

The electrochemical stability window for different PEG‐H_2_O‐NaCl mixtures in nitrogen (N_2_) and carbon dioxide (CO_2_) environments was determined using a three‐electrode cell configuration. The experiments utilized linear sweep voltammetry (LSV), which involved both anodic and cathodic sweeps ranging from +5 V to −5 V relative to silver (Ag), starting from the open circuit voltage (OCV). **Figure** **2** presents the results of all LSV measurements conducted for the PEG‐H_2_O‐NaCl mixtures in a N_2_ (Figure 2A) and in a CO_2_ atmosphere (Figure 2B). In both series, we observed a decrease in current during the cathodic sweep, initiating the potential onset at approximately −1.75 V. At this potential value, the electrochemical reduction of O_2_ in the electrolyte solution occurs first, followed by the formation of hydrogen gas from the reduction of H_2_O.^[^ ^25^ ^]^ In the anodic zone (from OCV up to 5 V vs Ag), the potential at which PEG oxidation began was observed at ≈1.45 V versus Ag for all electrolyte compositions. The presence of CO_2_ slightly shifted the onset potential for anodic oxidation by ≈50 mV toward lower values for most compositions compared to the electrolyte mixtures under N_2_. Given that the electrochemical stability of all the studied compositions had a water content ranging between 25 and 3 v/v%, we also examined the working window of an electrolyte composition with a 50:50 v/v% ratio of water to polymer (P50) in the presence of CO_2_ (Figure S7, Supporting Information). A notable narrowing of the electrochemical stability window by 1.80 V vs Ag was observed. This drop was attributed to the higher amount of “free” water in this formulation, a phenomenon previously observed in PEG electrolytes used in batteries.^[^ ^21^ ^]^ The high electrochemical stability observed in these PEG‐rich aqueous electrolytes can be attributed to water confinement through hydrogen bonding with the polymer. This is favored because alkyl groups in PEG donate electrons, making the ethereal oxygen atoms in PEG more electronegative than those in water. The mechanism that allows for a high electrochemical working window in these aqueous polymer electrolytes might help mitigate hydrogen and oxygen evolution reactions in the presence of CO_2_, preventing side reactions that could interfere with the supercapacitive capture. A closer examination of the onset points and values at which water reduction occurs is illustrated in the insets of Figure 2A and B. As the PEG content decreases, the reduction process shifts its onset potential to less negative values, particularly when the water content is higher. Interestingly, among all the samples studied, the behavior of those with the highest polymer content (P90, P93, P97) was very similar. This similarity may be attributed to the interactions between water molecules and between PEG and water present in the electrolytes. These interactions are closely related to the electrochemical stability window of the electrolyte samples and can alter their properties, impacting their capacity to maintain optimal performance in the CO_2_ capture process. Based on these results, we chose P75 and P90 as model electrolyte systems to study the SSA of CO_2_.

### 2.2 Thermodynamic and Kinetic Studies of SSA of CO_2_

A galvanostatic cycling limit potential (GCLP) method was implemented in a two‐electrode cell connected to a pressure sensor to evaluate the thermodynamic and kinetic performance of polymer electrolytes in the SSA of CO_2_. A comprehensive description of the measurement system that we used to monitor the adsorption and release of CO_2_ during the charging and discharging cycles, the components of the electrochemical cell, along with the explanation of the sequential steps for (de)gassing, is provided in the supplementary information file (Figure S8, Supporting Information). To preliminarily assess the impact of various electrochemical parameters on CO_2_ capture, we implemented both a positive charge protocol (PCP) and a negative charge protocol (NCP) for the electrolyte composition P90 (Figure S8A, Supporting Information). The experiments were performed across various voltage ranges, increasing by 0.5 V increments from +0.5 V to +2.5 V for the PCP (Figure S8B, C, Supporting Information) and from 0 V to −2.5 V for the NCP (see Figure S8D, Supporting Information). The experiments were carried out at a current density of 30 mA g^−1^, with holding times of 1 h and 4 h, and at a temperature of 40 °C.

**Figure 3:**
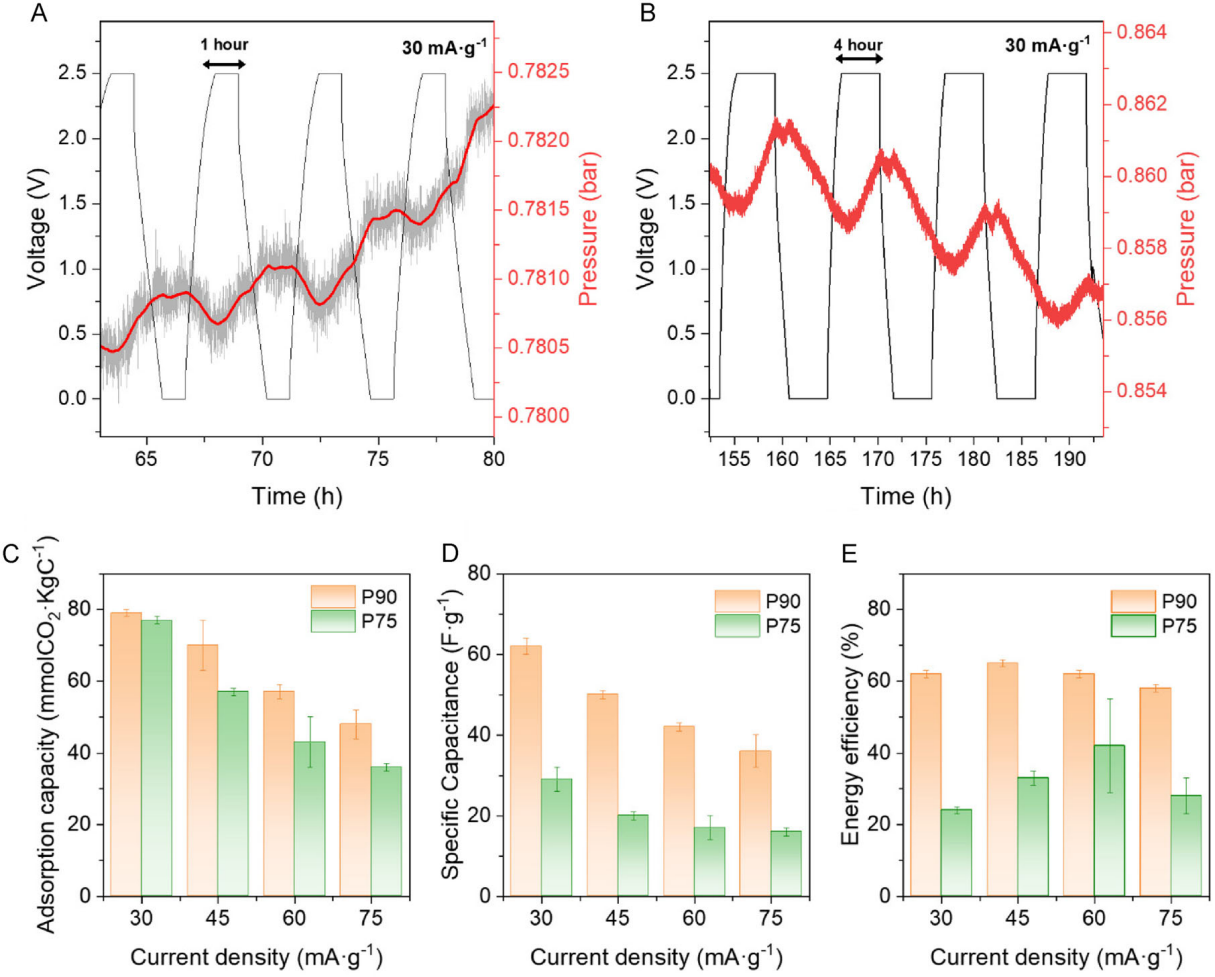
Comparison of the SSA of CO_2_ performance for P75 and P90 electrolytes, after subtraction of the contribution due to the progressive pressure drop in the system due to physical CO_2_ adsorption in the aqueous liquid polymer electrolyte. Zoomed area corresponding to a GCLP and pressure curves in a two‐electrode cell at a current density value of 30 mA g^−1^ within a voltage window between 0 and 2.5 V, and A) 1 h (smoothed pressure curve) and B) 4 h of holding time in the presence of a CO_2_ atmosphere for P90. Comparison of parameters between P75 (green) and P90 (orange) electrolyte composition: C) CO_2_ adsorption capacity, D) capacitance, E) energy efficiency.

According to the SSA mechanism of CO_2_ reported before,^[^ ^19^ ^]^ when applying a positive voltage (charge), an increase in pressure is expected due to the release of CO_2_, whereas when applying a less positive potential (discharge) a drop in pressure as a consequence of the adsorption of bicarbonate species in the gas‐exposed and positively charged electrode for the duration of the voltage cycle is likely to occur. A similar trend during the PCP was evidenced in our measurements using aqueous PEG‐based electrolytes. At positive voltage, no change in pressure was observed until the cell reached a voltage of 1.5 V (see Figure S8B, Supporting Information). A more significant variation in pressure was noted when the voltage increased to 2.5 V (see **Figure** **3**A). This exhibited a strong correlation between the electrochemical discharge/charge process and the supercapacitive capture/release of CO_2_ in the PCP after holding at that voltage for one hour. We observed a delay between the applied voltage and the pressure response. This delay is likely because the CO_2_ and bicarbonate ions move slowly during the one‐hour voltage hold.

We have also studied the SSA of CO_2_ for a negative cell voltage of −2.5 V by using an NCP (Figure S8C, Supporting Information), expecting an analogous behavior to that observed in the PCP^27^. However, the results differed now from PCP, and from those previously published based on SSA mechanism,^[^ ^11^ ^]^ evidencing an abrupt increase in the overall pressure during the charging process. After the implementation of an NCP, a PCP was applied again at a current of 30 mA g^−1^ in the voltage range between 0 V and 2.5 V. Like in the first PCP, it was found that, for a voltage value below 1.5 V, there was no appreciable variation in pressure. At 1.5 V and 2 V, a slight variation in pressure was observed, like the measurements taken before the NCP. To enhance the transport of species through the electrolyte, a positive charge of up to 2.5 V was applied, with a holding time of 4 h (Figure 3B). The results revealed a significant increase in pressure values after 4 h, showing a 600% enhancement compared to the 1‐hour holding period. This indicates a higher efficiency in CO_2_ capture under these conditions. Furthermore, there was no delay in the pressure response during the voltage hold time, which improved the adsorption capacity, as the ions could respond promptly to changes in cell voltage.

We have also observed a progressive drop with time in the overall pressure value (Figure S8, Supporting Information). In order to determine if the continuous decrease of pressure during the charging and discharging processes was due either to a corrosion phenomenon^[^ ^19^ ^]^ or to a physical adsorption of CO_2_ within the aqueous polymer electrolyte, we ran a measurement where the pressure value was initially recorded only under OCV conditions for a period of time enough to assure the diffusion of the chemical species from the electrolyte, including the CO_2_, to the surface of the electrode before apply any current (Figure S9, Supporting Information). The results evidenced a constant drop in pressure during the OCV as well as when the cell was subsequently charged (oxidized) and discharged (reduced) by GCLP at a current density of 30 mA g^−1^ up to 2.5 V with a holding time of 4 h. This variation in pressure may prove the most probable simultaneous physical adsorption of CO_2_ in the electrolyte system and probably also in the pores of the carbon electrode material as bicarbonate ions, along with the SSA of CO_2_, and discard the possibility of having a corrosion process. This observation is further supported by the pressure drop we still noticed when cycling within a voltage range of 0 V to 0.5 V. This suggests that the decrease in pressure values during cycling is not due to a corrosion reaction from an electrochemical process.

To confirm this fact, we also performed a measurement only under nitrogen gas atmosphere, using the same current density values of 30 mA g^−1^, voltage window of 2.5 V, and a holding time of 4 h (Figure S10, Supporting Information). We observed a very slight variation of the cell pressure corresponding to a capture of 10 mmol kg^−1^, which can be mostly attributed to dimensional changes of the microporous electrodes during the charge and discharge in the supercapacitor.^[^ ^26^ ^]^ Because of this fact, and to be as accurate as possible on the quantification of the amount of CO_2_ captured electrochemically, we removed the non‐SSA of CO_2_ contribution from our measurements by processing data using MATLAB (see Figure S3, Supporting Information).

Based on the preliminary results, the optimal conditions for studying the thermodynamic and kinetic properties and quantifying the amount of CO_2_ captured by SSA using a PEG‐based electrolyte system, while applying a galvanostatic method, appear to be a potential window between 0 V and ±2.5 V, a holding time of 4 h, and a constant temperature of 40 °C. We chose the electrolyte compositions P75 (Figure S11, Supporting Information) and P90 (Figure S12, Supporting Information) to conduct a more in‐depth study on the SSA of CO_2_, and then we discussed the findings (Figure 3C). Our goal was to investigate the relationship between the amount of CO_2_ adsorbed using each electrolyte (Figure 3C) and the electrochemical capacitance of the electrodes (Figure 3D). Additionally, we aimed to evaluate the energy efficiency of the cell at various current densities of 30, 45, 60, and 75 mA g^−1^ (Figure 3E) (NOTE: when applying a current density value of 15 mA g^−1^, we did not observe adsorption of CO_2_, most probably due to the high kinetic restrictions at that low flow of charges. It is also possible that some reversibility occurs. In general, both electrolyte compositions (Figure S11 and S12, Supporting Information) exhibited a similar behavior, with overall pressure drops attributed most probably to a physical adsorption of the CO_2_‐derived species. It was also evident that the overall slope from the pressure drop line for the P90 composition is significantly higher than for P75. This behavior could be attributed to the higher content of polymer with respect to water, present in P90, which could favor a higher physical adsorption amount of CO_2_ in the electrolyte. However, the maximum values of the adsorption capacity of CO_2_ evidenced for both compositions P75 and P90 at 30 mA g^−1^ during the first PCP were roughly similar, 77 mmol CO_2_ kg^−1^ and 79 mmol CO_2_ kg^−1^, respectively (Figure 3C). Although a more marked difference was observed in favor of the P90, which showed higher adsorption values compared to P75 at higher current densities, a progressive drop in the CO_2_ adsorption capacity with the current density for both compositions was evidenced. The analysis of the results of the SSA of CO_2_, obtained at various current densities, regardless of the electrolyte composition studied, confirms that the CO_2_ capture mechanism is primarily a kinetic effect. This process becomes reversible at an optimum current density of 30 mA g^−1^ at which the transport of CO_2_ and bicarbonate ions is less constrained.

Regarding the capacitance value per electrode, a significant difference between the two electrolytes was revealed (Figure 3D). At 30 mA g^−1^, and during the first PCP, P75 presented a capacitance value of 29 F g^−1^, while P90 reached a considerably higher value of 62 F g^−1^. At higher current densities, the capacitance decreases for both electrolytes, but the capacity retention values for P90 were consistently higher than for P75, suggesting that the higher proportion of PEG in P90 contributes to a better storage capacity compared to P75. The observed results may be attributed to differences in ion adsorption efficiency on the carbon surface across, depending on the polymer electrolyte composition. This efficiency is influenced by the amount of “free water” molecules present, which affect two key parameters: 1) the solvation shell of the ions and 2) the “wettability” and accessibility of the electrolyte to the carbon surface (e.g., pores). Related to the former, as the amount of “free water” in the electrolyte decreases, as seen in P90 compared to P75 in Figure 1 and 2, the probability that ions will adsorb in a desolvated or dehydrated state within the pores increases. This process allows the ions to come closer to the electrode surface. As a result, it leads to more effective charge screening and storage, ultimately resulting in increased capacitance.^[^ ^27^, ^28^ ^]^ Regarding the latter, a higher concentration of polymer in the electrolyte may increase its affinity for the electrode's surface due to the hydrophobic characteristics of the carbon material. Then, the carbon structure and its defects could drive denser ion packing in the pores, which could lead to improved capacitive performance, as has been recently hypothesized.^[^ ^29^ ^]^ In any case, these results emphasize the significant influence of polymer electrolyte composition on the electrochemical response of the system. Among the formulations tested, P90 demonstrates the highest capacitance retention and the largest amount of CO_2_ adsorbed per kilogram of electrode across the entire range of current densities examined. In terms of energy efficiency (see Figure 3E), P90 maintains a steady energy efficiency value of ≈60% across all current densities studied. In contrast, P75, at a current density of 30 mA g^−1^, exhibits a notably lower energy efficiency of 25%.

**Figure 4:**
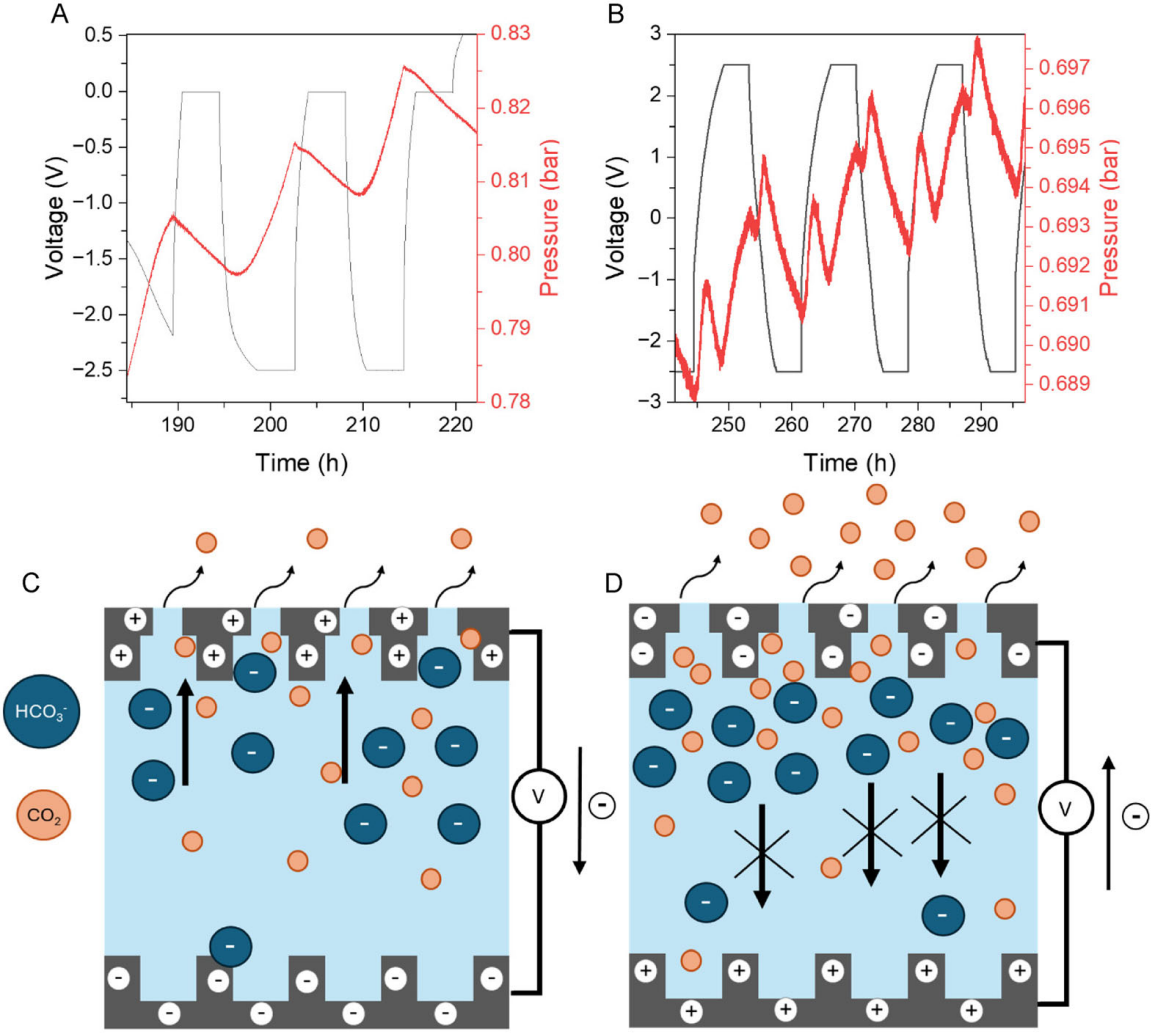
Zoomed area corresponding to a GCLP and pressure curves in a two‐electrode cell at a current density value of 30 mA g^−1^, 4 h of holding time within a voltage window between A) 0 and −2.5 V, B) 2.5 V and −2.5 V, and a current density of 30 mA g^−1^ in the presence of a CO_2_ atmosphere for composition P90. Schematic showing the proposed mechanism for the movement of CO_2_ and HCO_3_^−^ in the C) positive charging protocol (voltage window: 0 to 2.5 V) and D) negative charging protocol (voltage window: 0 to −2.5 V).

The study on the SSA of CO_2_ conducted at a negative cell voltage value of −2.5 V for the compositions P75 (Figure S11C, Supporting Information) and P90 (**Figure** **4**A), demonstrated a rapid increase in pressure during the charging process, followed by a decrease during discharge. This behavior contrasts with what was observed while using a PCP and differs from results reported in previous studies based on the SSA mechanism.^[^ ^11^ ^]^ This finding indicates that the CO_2_ capture and release mechanism behaves similarly to a symmetric system, exhibiting no dependence on the direction of the charge. Interestingly, applying NCP up to −2.5 V increased the amount of CO_2_ adsorbed per kilogram of electrode up to 356 mmol CO_2_ kg^−1^ with a capacitance of 42 F g^−1^ and energy efficiency of 27% for P90 and up to 202 mmol CO_2_ kg^−1^ with a capacitance of 9 F g^−1^ and an energy efficiency of 7% for P75. The effect of the symmetric capture becomes even more evident when a switching protocol using the full range of voltage window from −2.5 V to +2.5 V was applied (Figure 4B and S13, Supporting Information). The pressure data presented in Figure 4B demonstrates two cycles of capture and release processes. This pattern likely occurs as follows: during the discharge process from −2.5 V to 0 V, CO_2_ is captured. Then, during the charging phase from 0 V to +2.5 V, the CO_2_ is released. After holding the voltage for 4 h, the electrode discharges again from +2.5 V to 0 V, capturing CO_2_ for a second time, followed by a final charge from 0 V back to −2.5 V, which releases CO_2_ once more. Previous published works by the Forse group on SSA of CO_2_ have suggested that the adsorption is most probably driven by the concentration of bicarbonate ions near the gas‐exposed electrode material.^[^ ^11^ ^]^ Our results suggest that the enhancements observed during the NCP in comparison with the PCP (Figure 4C) may be due to two factors: the ejection of pre‐adsorbed bicarbonate species from the pores^[^ ^30^, ^31^ ^]^ and their slow migration kinetics toward the positively charged counter electrode (CE) under CO_2_ gas exposure, as shown in Figure 4D.

Subsequently, upon reestablishing positive polarity and performing a second charging of the cell from 0 V to 2.5 V by means of a new constant potential charging, PCP, cycle, the same electrochemical behavior described during the first PCP was again observed (Figure S11D and S12C, Supporting Information). However, during this second stage, a decrease in the amount of adsorbed CO_2_ was evidenced (31 mmol CO_2_ kg^−1^ and 68 mmol CO_2_ kg^−1^, for P75 and P90 respectively at 30 mA g^−1^), as well as in the capacitance values (20 F g^−1^ and 50 F g^−1^, P75 and P90 respectively at 30 mA g^−1^) and energy efficiency values (25% and 50% P75 and P90 respectively at 30 mA g^−1^) for both electrolyte compositions compared to those obtained during the first PCP. For the P75 composition, although a lower amount of adsorbed CO_2_ is also recorded by SSA after the polarization change, a significant increase in the ohmic resistance of the cell was also detected. This increase in resistance complicates the accurate evaluation of parameters such as capacitance, coulombic, and energy efficiency, due to the alteration in the overall electrochemical response of the system. Overall, the amount of CO_2_ captured as well as the specific capacitance and energy efficiency present higher values before the polarization change, suggesting a possible partial degradation of the performance after successive polarity reversal cycles.

**Figure 5:**
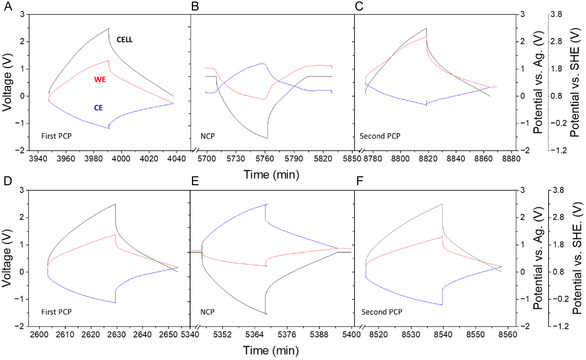
Three‐electrode experiment of P90 (top) and P90MB (bottom). The positive charging protocol A,D), voltage window 0 V–2.5 V; negative charging protocol B,E), voltage window 0 V–−2.5 V; second positive charging protocol C,F), voltage window 0 V–2.5 V. The potential variation of the full cell (black), the WE (red), and CE (blue) in a GCLP measurement is also shown; current density, 30 mA g^−1^. The potential variation of WE and CEs is represented versus Ag and Standard Hydrogen Electrode reference electrodes.

To better understand the operating mechanism of our electrochemical cells, we used a silver rod as a pseudoreference electrode. This allowed us to gather additional information about the capacitance and the actual operating potential of each electrode during its operation: the CE (located far from the CO_2_ gas reservoir) and the working electrode (WE) (located near the CO_2_ gas reservoir). We examined both P90 (**Figure** **5**) and P75 (Figure S14, Supporting Information) compositions in our study.

The P90 composition (Figure 5A–C) presented balanced charge and discharge processes during the first PCP, reflecting an ideal electrochemical double‐layer behavior. In this case, the potential values for the WE and the CE were 1.21 V and 1.29 V versus Ag, respectively, indicating similar capacitance values in both electrodes. The slightly more negative OCV value might suggest a more negative surface charge on the WE due to the specific interactions between the anions and the carbon surface. The electrochemical results show a similar situation for the negative polarization of the cell (WE: 1.37 V vs Ag; CE: 1.09 V vs Ag), but this time, both electrode materials showed a strong interaction for the ions oppositely charged (Figure 5B). This is consistent with the presence of a charge storage mechanism (including the bicarbonate anions derived from CO_2_) in the electrochemical double‐layer capacitors involving the possibility of electrostatic counter‐ion adsorption, co‐ion desorption, and counter‐ion*‐co*‐ion exchange,^[^ ^30^ ^]^ where the bicarbonate anions are desorbed from the gas‐exposed carbon electrode while at the same time the sodium cations are adsorbed. On the opposite side of the cell, bicarbonate anions are being attracted to the CE surface, while the chloride anions are most probably being expelled from them. Such a difference in the ion fluxes connected to the change in the OCV value in both electrodes is important for the supercapacitive storage mechanism, as it may affect not only the kinetics of the charge storage mechanism but also the kinetics of the SSA of CO_2_. The second PCP (Figure 5C) shows a different behavior; the WE operates at a potential twice as high as the CE (WE.1.66 V CE. 0.81 V), losing the cell symmetry, which could indicate the decrease of the parameters obtained in the second PCP compared to the first one. In the cell containing the electrolyte with P75 composition, prior to the polarity change (see Figure S14A, Supporting Information), it was observed that the charge process took significantly longer than the discharge process. In this case, the WE and CE were operated at potentials of 1.20 V and 1.30 V versus Ag, respectively. Following the polarity change, there was a noticeable sharp increase in ohmic resistance in the P75 composition (refer to Figure S14B, Supporting Information), which resulted in significantly reduced electrode performance compared to the P90 composition.

Seeking to improve the properties of P90 and due to the fact that the WE was operational in a significantly larger potential window compared to the CE and therefore the capacitance of the latter was significantly lower than the CE, a new measurements was performed in a two‐electrode cell system, adjusting the mass ratio between the WE and CE of 2.2:1 (Figure S15, Supporting Information). The overall electrochemical behavior of the cell, now called P90MB (MB: mass balance), remains like the previous experiments (Figure S15A, Supporting Information). During PCP (Figure S15B, Supporting Information), an increase in pressure is observed during cell charging and a decrease in pressure during cell discharge, and in NCP (Figure S15C, Supporting Information), a rapid increase is observed during cell charging and a decrease during cell discharge, while maintaining a symmetry comparable to previous measurements was detected. However, the values obtained for CO_2_ capture (40 mmol CO_2_ kg^−1^), capacitance (36 F g^−1^), and coulombic efficiency (50%) at 30 mA g^−1^ before the first polarization change were significantly lower than those recorded in experiments without the MB of the electrodes. During the NCP, the electrochemical adsorption of CO_2_ reached 110 mmol kgC^−1^, tripling the amount obtained during the polarization of the cell using a PCP with a capacitance of 7 F g^−1^. This confirms the same behavior observed in previous cases using the NCP. Moreover, these results suggest that the electrochemical CO_2_ capture and capacitance are not directly related, existing other factors influencing the SSA of CO_2_ mechanism. Subsequently, the cell was positively charged again at 2.5 V (Figure S15D, Supporting Information), at different current densities, showing the same behavior as in the first PCP. The values of the parameters studied (C_CO2_ = 29 mmol kgC^−1^, C = 28 F g^−1^) were lower than those from the first PCP, as in the system without the MB of the electrodes.

To evaluate the impact of MB on the electrochemical behavior of the cell and to investigate in more detail the mechanism of SSA of CO_2_ and the potential at which the electrodes operate, the previous experiment was also performed with a silver pseudoreference electrode (Figure 5B). In the first charge‐discharge cycle (PCP) (Figure 5D), it was observed that the charge process presented a slightly longer duration than the discharge process, a phenomenon that was not evident in previous experiments with P90 without the MB of the electrodes. Nevertheless, both electrodes showed adequate electrochemical behavior, operating at potentials of 1.20 V and 1.30 V for the WE and CE, respectively; these values were consistent with previous experiments without MB. After reversing the cell polarity (Figure 5E), an increase in the ohmic resistance of the electrodes and an inhomogeneous distribution of the cell potential were detected, with values of 0.76 V and 1.76 V vs Ag for WE and CE, respectively, indicating a deterioration in performance compared to the configuration without MB. Next, by positively recharging the cell to 2.5 V (Figure 5F), the potential at which the electrodes work showed more similar values than in the NCP and the second PCP of the composition without MB, operating at 1.11 V and 1.41 V versus Ag for WE and CE, respectively, although with some increase in the ohmic resistance. Although the potential symmetry in the cell improved, there were no observed enhancements in either the amount of CO_2_ adsorbed or the cell's capacitance. This suggests that other factors, such as the composition of the electrolyte or the structure of the carbon porous material, may significantly impact the system's performance. In summary, while the MB of the carbon electrode enhances the system's capacitive properties, the kinetics of CO_2_ adsorption are still influenced by ion transport across the electrolyte and potentially competing reactions at both electrodes.

To evaluate the potential use of these aqueous‐based polymer electrolytes in industrial processes that involve exhaust vents containing CO_2_ and other gases such as O_2_, a study was conducted using a gas mixture of 50% v/v CO_2_% and 50% v/v air (see Figure S16, Supporting Information). This assessment focused on the optimized aqueous polymer electrolyte formulation P90 (refer to Figure S16A, Supporting Information). The operational conditions included applying both a PCP (Figure S16B, Supporting Information) and an NCP (Figure S16C, Supporting Information) across a voltage range of 0 V to ±2.5 V, at a temperature of 40 °C. The results were quite remarkable, demonstrating CO_2_ adsorption capacities of up to 36 mmol CO_2_ per kg of carbon (kg C^−1^) at a current of 30 mA g^−1^ when using a PCP, and when the NCP was applied, the capacities reached 180 mmol CO_2_ kg C^−1^ at a current density of 30 mA g^−1^ and 115 mmol CO_2_ kg C^−1^ at a current density of 45 mA g^−1^.

**Figure 6:**
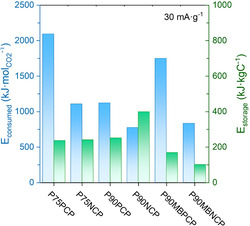
Comparison between compositions P75, P90, and P90MB in the Positive charging protocol (0 to 2.5 V) and the negative charging protocol (0 to −2.5 V) at 30 mA g^−1^. In blue specific energy consumed per mole of CO_2_ captured and in green cell energy stored per kilogram of electrode.

One of the key aspects of this study is the broad potential window offered using polymer electrolytes. As a result, while other systems^[^ ^14^ ^]^ may exhibit higher capacitance values, their energy storage capacity is significantly restricted when operating within relatively low potential windows (between 0.8 V and 1.3 V). In our case, thanks to the high electrochemical stability of the polymer electrolyte used, it is possible to operate safely in a potential window of up to 2.5 V. Since the energy stored in a supercapacitor is quadratically related to the operating voltage (*E* = 1/2 *C* *V* ^2^), this increase in the potential window makes it possible to achieve stored energy values up to an order of magnitude higher. This result underlines the importance of developing stable electrolytes over a wide range of potentials, since even with similar capacities, the impact on the overall energy yield can be significantly higher. In this context, the energy consumed and energy stored per mole of CO_2_ captured were compared for four different compositions: P75, P90, and P90 with MB (P90MB) in their positive/negative charging protocol at 30 mA g^−1^. **Figure** **6** shows the comparison of the specific energy consumed per mole of CO_2_ captured (blue bars) and in the cell energy stored per kilogram of electrode (green bars) at a current density of 30 mA g^−1^. It is observed that composition P90 NCP has the lowest energy consumed and the highest stored energy of all the compositions, despite having a low energy efficiency. On the other hand, P75 PCP has the highest energy consumption per mole of CO_2_ adsorbed and low energy storage. This behavior is attributed to the high polymer content in the electrolyte compositions, which increases the viscosity of the medium and, consequently, the resistance to ionic transport, increasing the energy cost per mole of CO_2_ captured. However, this disadvantage is partially compensated by the stored energy, favored by the wide operating potential window of the system.

Conventional amine‐based CO_2_ separation processes typically require around 100 kJ mol^−1^ of CO_2_, with reported maximum energy efficiencies of ≈21%.^[^ ^6^, ^7^ ^]^ In contrast, recent studies on SSA have demonstrated energy consumption as low as 20 kJ mol^−1^ of CO_2_ with higher energy efficiency, highlighting the potential of this emerging technology.^[^ ^1^ ^]^ Because SSA is still a technology under development, frequently the capture metrics that scientists compare with other carbon capture technologies are based on the amount of moles of CO_2_ captured divided by the mass of electrode material. Herein, we show a different strategy based on the use of polymer electrolytes to further reduce the energy consumption, achieving energy efficiencies greater than 60%, which underscores the promise of our approach to be implemented in the future as a low‐energy CO_2_ capture technology.

## 3 Conclusion

A systematic evaluation of a series of aqueous polymeric electrolytes formulated to operate at neutral pH conditions has been carried out, which has demonstrated remarkable electrochemical stability and a wide voltage window, key parameters in the application of the SSA technique for CO_2_ capture. The electrolytes studied have shown great resistance to electrochemical degradation even under high voltages and during prolonged periods of charge maintenance. Also, no signs of corrosion have been observed on the electrodes during cell operation, indicating good compatibility between the electrolytic system and the electroactive materials used. These electrolytes have allowed the successful exploration of negative charge strategies, i.e., potential sweeps from 0 to −2.5 V, resulting in a substantial improvement in CO_2_ adsorption capacity. This phenomenon could be attributed to the high local concentration of bicarbonate ions (HCO_3_^−^) in the proximity of the electrode exposed to the gas, an accumulation favored by the slow kinetics of ionic transport and adsorption. This mechanism suggests a charge direction‐independent behavior, which deserves further study.

One of the most significant findings of the study is that using negatively charged protocols enhances CO_2_ capture capacity. Furthermore, in the case of the P90 NCP composition, a notable decrease in energy consumption was observed compared to the other compositions evaluated. This energy performance, along with a high adsorption capacity (up to 356 mmol of CO_2_ per kilogram of electrode), positions this aqueous‐based polymer electrolyte system as a particularly promising option for future use. The results highlight the potential of these safe and sustainable polymer electrolytes as a viable alternative for electrochemical gas capture technologies. Further experiments are needed to understand the adsorption mechanism at the molecular level and to optimize the energy efficiency of the electrochemical CO_2_ capture process.

## Conflict of Interest

The authors declare no conflict of interest.

## Supporting information

Supplementary Material

## Data Availability

The data that support the findings of this study are available from the corresponding author upon reasonable request.
