# Supplementary material for: Supercapacitive CO2 Capture through a Scalable, Aqueous, Neutral Polymer‐Based Electrolyte
Source: ChemSusChem. 2025 Sep 22;18(22):e202501259. doi: 10.1002/cssc.202501259 (PMC12642970; doi:10.1002/cssc.202501259)
Supplement: Supplementary file 1 — Supplementary Material [file CSSC-18-e202501259-s001.pdf]

Supercapacitive CO_2 capture through a scalable, aqueous, neutral polymer-based electrolyte

Daniel García-Giménez, Marta Santos-Rodríguez, Antoniou Maria-Anna, Mohammad Sanan-Ali, Miguel A. López-Manchado and Javier Carretero-González*

[a] Daniel García-Giménez, Marta Santos-Rodríguez, Antoniou Maria-Anna, Mohammad Sanan-Ali, Miguel A. López-Manchado J. Carretero-González

Institute of Polymer Science and Technology, ICTP, CSIC, 28006, Madrid, Spain

E-mail: jcarretero@ictp.csic.es

SUPPORTING INFORMATION

EXPERIMENTAL SECTION.

MATERIALS. Polyethylene glycol 400 (PEG), sodium chloride (NaCl) and deuterium oxide (D_2O_2) were purchased from Sigma Aldrich and used as it received. Aqueous electrolyte solutions were prepared using deionized water (15 mOhm). The carbon electrodes were prepared by using microporous active carbon YP-80F (Kuraray), and an aqueous colloidal suspension (60 wt. %) of polytetrafluoroethylene (PTFE) from Sigma Aldrich, as a binder material.

ELECTROLYTE PREPARATION. For the preparation of the polymeric electrolyte formed by PEG-H_2O-NaCl, the NaCl salt was first dissolved in deionized water up to a concentration value of 0.2 M for NaCl in the final electrolyte. Then, both aqueous solutions are mixed, and homogenized by stirring. Subsequently, the solution is left to rest for 1 hour.

VISCOSITY, PH AND IONIC CONDUCTIVITY. A Brookfield Ametek DVnext viscometer was used to measure viscosity at 40°C and 200 rpm. The pH and ionic conductivity of the compositions were measured with a Thermo Fisher Scientific Eutech PC 2700 tester. Prior to measurement, pure CO_2 streams were introduced to all compositions for at least 30 minutes.

DIFFERENTIAL SCANNING CALORIMETRY (DSC) ANALYSIS. Thermal analysis of PEG 400 and H_2O mixtures was performed with Differential Scanning Calorimetry DSC 214 Polyma (DSC) under an Air and CO_2 atmosphere. The experiments used 15 mg for each sample in closed capsules in cooling and heating cycles of 10°C/min from 60ºC to -90ºC and from -90°C to 60°C, respectively.

NMR EXPERIMENTS. The ^1H NMR analyses were carried out with the BRUKER AVANCE III-400 and specific liquid and gas tubes (Norell™ Borosilicate Glass J Young NMR Valves), and branded deuterated water was used under N_2 and CO_2 atmosphere.

For the sample, the mixture of PEG and deuterated water was prepared by bubbling gas and then introduced into the tubes for measurement.

ELECTRODE PREPARATION. To prepare YP-80 electrodes, we employ a lamination method. The composition of the electrodes includes 95% YP-80F, 5% PTFE (60%), and ethanol as a solvent. All components were mixed and stirred until the ethanol is nearly completely evaporated. The mixture was then transferred to a watch glass until a viscous mass forms. This mass was subsequently moved to a glass laminate and kneaded several times with a glass tube until a laminate of uniform thickness was achieved. Finally, the laminate was placed in an oven at 90ºC for 24 hours to remove any remaining water and ethanol traces. The electrodes were then cut to a diameter of 11 mm for use in two-electrode electrochemical measurements.

ELECTROCHEMICAL MEASUREMENTS*.* Electrochemical analysis was conducted using a VMP3 multichannel potentiostat (Biologic). The electrochemical stability of the mixtures of water and PEG were assessed through Linear Sweep Voltammetry (LSV), utilizing a three-electrode setup. The voltage was scanned within a range of -5V to 5V vs Ag at a rate of 1 mV/s. A glassy carbon electrode, with a diameter of 3 mm, served as the working electrode; a silver rod acted as the reference electrode; and a platinum wire was employed as the counter electrode. To create an atmosphere of N_2 or CO_2, gas was bubbled through the polymer electrolyte for at least 30 minutes, and this atmosphere was maintained over the surface of the liquid during the measurement without any gas interference.

CO_2 CAPTURE EXPERIMENTS*.* A system was designed to monitor the capture or adsorption of CO_2 (see Figure S1). This system consists of a series of valves connected to a reservoir with a known gas volume. It includes a two-electrode cell equipped with a pressure sensor that measures pressure fluctuations within the cell. In the two-electrode cell, there were two YP-80F electrodes in contact with a solid and a hollow titanium plunger serving as current collectors in a symmetric cell configuration (Figure S2). The electrode near the hollow plunger is the working electrode and is on top of a titanium mesh to ensure better contact with the current collector. The bottom electrode is the counter electrode and is directly in contact to the surface of the titanium

plunger. The electrodes are separated by a glass fiber membrane (13 mm in diameter) that holds 400 µL of a polymer electrolyte solution (Figure S2A). For measurements, the cell was first subjected to slight vacuum conditions, after which a known volume of gas was introduced. This process was repeated five times, reaching a pressure of approximately 750 mbar. The cell was then placed in an incubator set at 40ºC. The experimental study used galvanostatic measurements at various current densities and voltage windows, implementing a positive/negative charging protocol adapted from Zhu et al.[1] Before starting the experiments, the supercapacitor cell underwent a series of cyclic voltammetries to equilibrate the system (Figure S2B).

To calculate the specific capture of CO_2 (in mmol $CO_2 \cdot kg^{-1}$), a model was employed that accounts for the pressure changes throughout the entire system. The simulation parameters were tailored to the specific system being developed. The number of moles of CO_2 gas was determined using the ideal gas law.

$$n = \frac{P \cdot V}{R \cdot T} \qquad (eq. S1)$$

The R value represents the ideal gas constant, while P, V, and T denote the pressure (in atm), volume (in L), and temperature (in K) of the system at each point, respectively. The total volume of the cell is derived from a known volume of the system that has been previously calibrated. This is done by using the equation $P_1 \cdot V_1 = P_2 \cdot V_2$, which considers the various pressure values measured during the (de)gassing process.

To account for variations in pressure data caused by signal oscillations during experimental measurements and pressure losses in the system due to external factors (such as physical CO_2 adsorption), the variation in the number of moles for each interval is calculated by using the maximum and minimum values observed in each cycle.

$$\Delta n_{average} = \frac{1}{N} \cdot \sum_{i=0}^{i=N} n_{max,i} - n_{min,i} \qquad (eq. S2)$$

Where N represents the total number of cycles n_{max} and n_{min} the maximum and minimum values of each interval. To reduce noise in the experimental pressure data, a Gaussian filter is applied for smoothing. Any irreversible drop in the signal is addressed using a mathematical model developed with Matlab® software. This process allows us to correct the data trend and produce a uniform dataset (see Figure S3).

The specific adsorption capacity of CO_2 ($mmol_{CO2}$·kgC^{-1}) of the system is calculated based on the difference between the initial and final number of moles in the system:

$$C_{CO_2} = \frac{\Delta n_{\ average}}{m}\ 10^6 \qquad (eq. S3)$$

Where C_{CO2} represents the captured CO_2 and m denotes the active mass of the working electrode in kilograms.

To gain a better understanding of these experiments and to analyze the behavior of the cell before and after the polarization change, we conducted measurements using a three-electrode system for both compositions. These measurements enabled us to evaluate the individual performance of each electrode during the electrochemical process. For that, a Swagelok T-shaped cell (see Figure S4) was used, featuring two titanium current collectors. The top current collector contains YP80 (WE) along with a titanium mesh to enhance the contact between the electrode and the current collector. In the lower current collector (CE), another YP80F electrode is placed. A silver bar, sealed to prevent gas leakage, was employed as the reference electrode. Glass fiber serves as a separator between the two YP80 electrodes. Approximately 400 µL of electrolyte was introduced into the cell to ensure that it wets both electrodes. The study is conducted using a GCLP of 30 mA/g.

Supplementary methods

EQUATIONS

The specific capacitance (F g^{-1}) was calculated from the discharge on the galvanostatic curves of the CO_2 capture experiments using the equation as we have non-linear GCLP profile:

$$C_{electrode} = 2\frac{I\int_{t_1}^{t_2} V\,dt}{m\,\Delta U} \qquad (eq. S4)$$

Where I, the applied current (A), Δt, the time it takes to discharge (s), m, the active mass of the working electrode (g), ΔU, the cell voltage deleting the cell resistance (V).

The specific energy consumed (Ed, kJ mol_{CO2}^{-1}) has been calculated with the following equation:

$$E_d = \frac{(\frac{I}{m}\int_{t_1}^{t_2} U_c\,dt - \frac{I}{m}\int_{t_1}^{t_2} U_d\,dt)}{C_{CO2}} \cdot 10^3 \qquad (eq. S5)$$

Where I, is the current intensity used during cell charging/discharging (A), m is the mass of the working electrode (g), U(t) is the cell voltage that varies with time during cell charging/discharging (V), t_1, is the beginning of charging/discharging (s) and t_2, is the end of charging/discharging (s), C_{CO2} is the CO_2 adsorption capacity. The energy stored in the supercapacitor per kilogram of electrode when we have non-linear GCLP profile is:

$$E = \frac{I}{m}\int_{t_1}^{t_2} U_c\,dt \qquad (eq. S6)$$

Where I, is the current intensity used during cell charging/discharging (A), m is the mass of the working electrode (g), U(t) is the cell voltage that varies with time during cell charging/discharging (V)

Supplementary figures

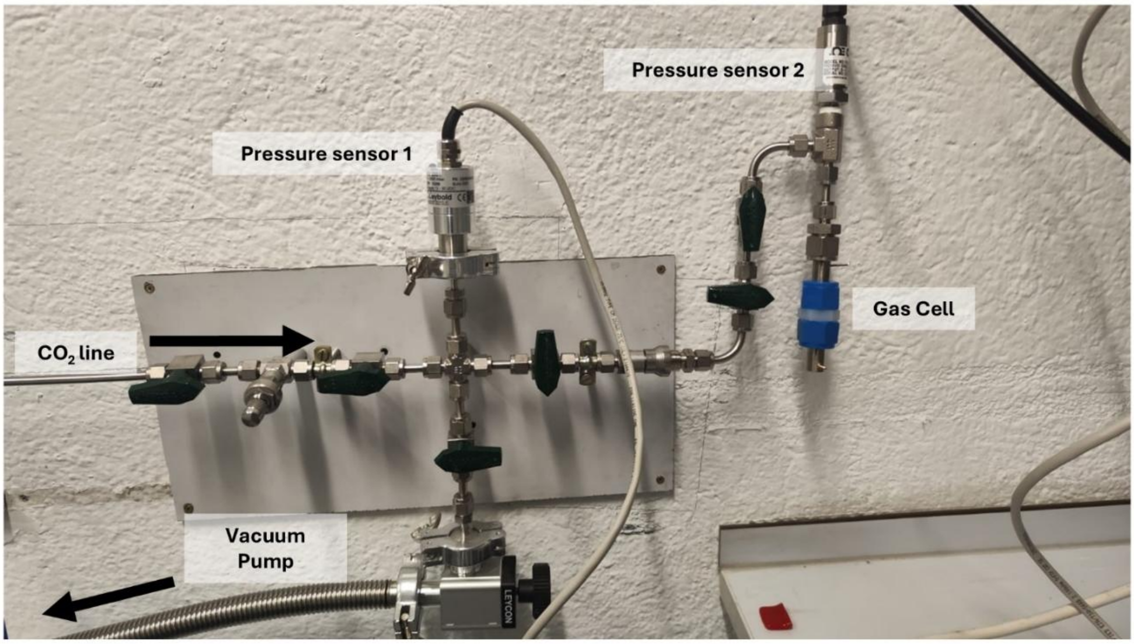

Figure S1. System for the introduction of CO_2 or N_2 in the cell, with the pressure sensor 1. We control the gas that we introduce in the cell, and with the pressure sensor 2, we monitor the pressure changes inside the cell.

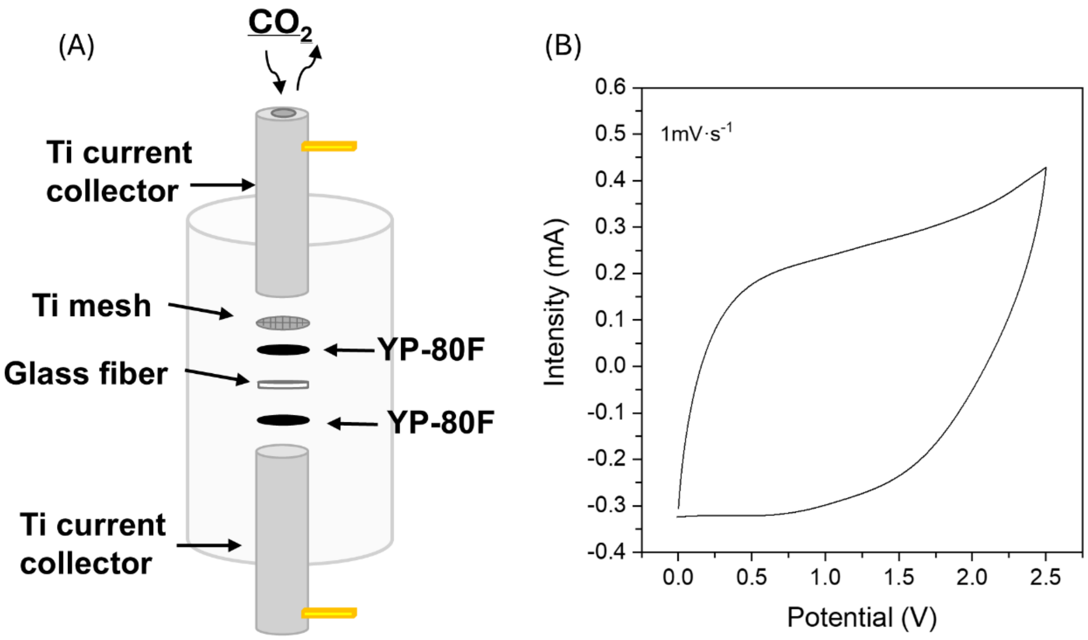

Figure S2. (A) Schematic of the Swagelok cell in 2 electrodes. (B) CV of P90 composition in 2 electrodes Swagelok cell at 1 mV·s^{-1} from 0 to 2.5 V, show rectangular and symmetric CV plots characteristic of a capacitive charge storage mechanism (double-layer charge/ discharge).

(A)

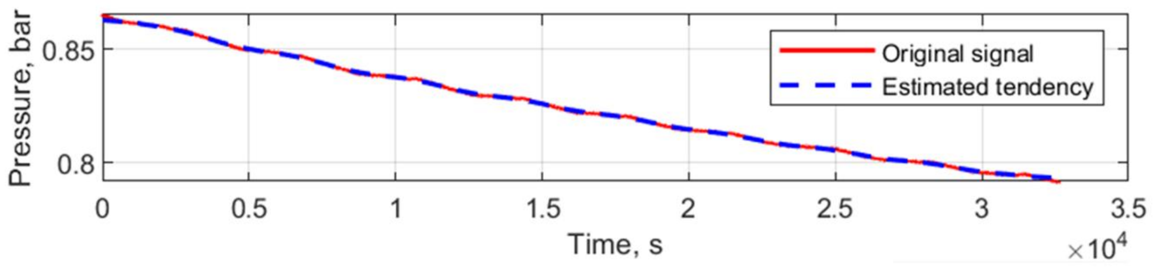

(B)

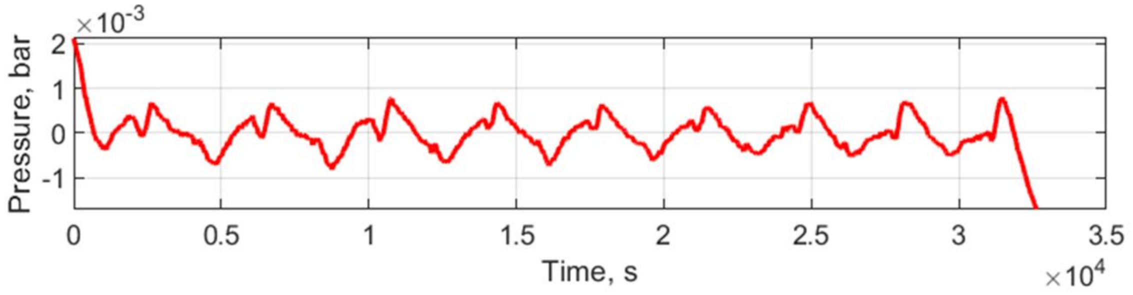

Figure S3. Adjustment of general decrease in a pressure curve using a mathematical model using Matlab software. (A) Red line, original signal of pressure; blue, estimated tendency the software will use. (B) Pressure curve adjusted without the general decrease.

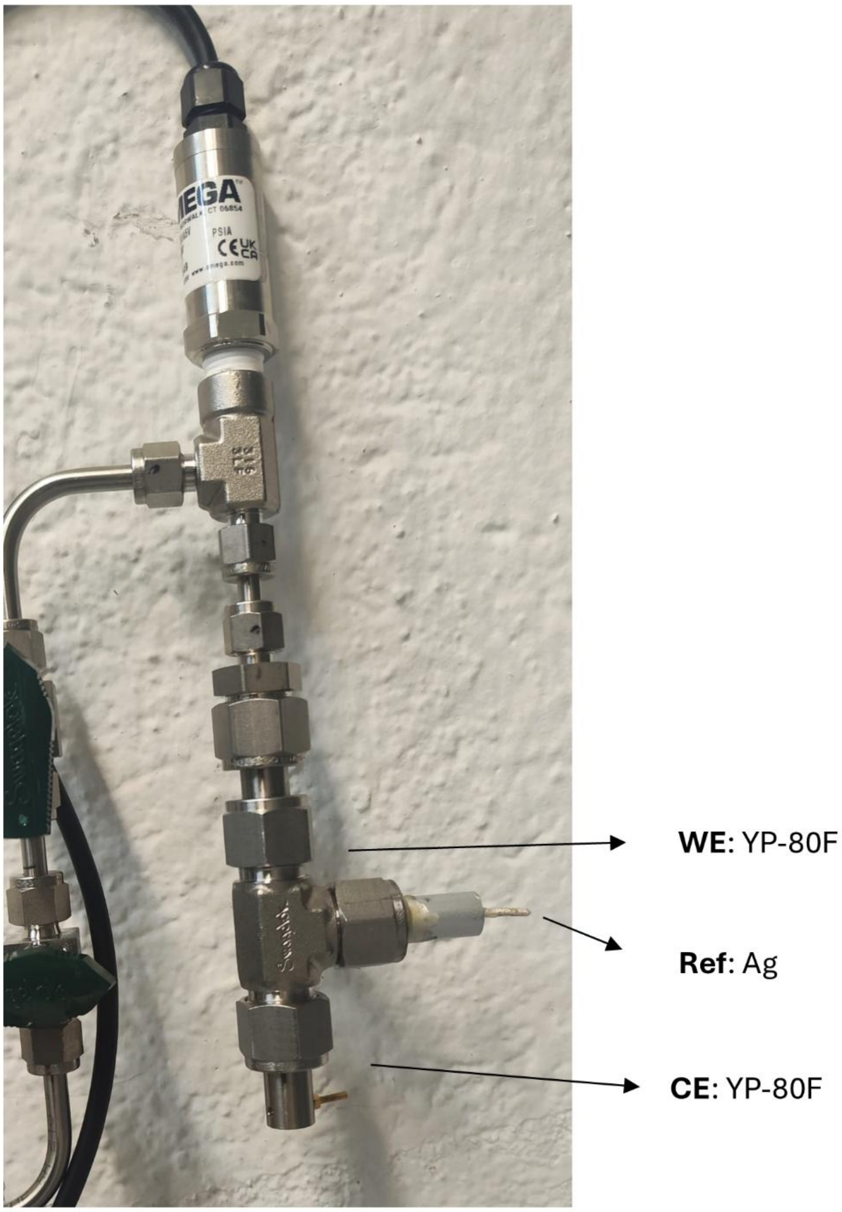

Figure S4. Three-electrode Swagelok cell, with WE and CE, YP-80F carbon; reference electrode, Ag wire.

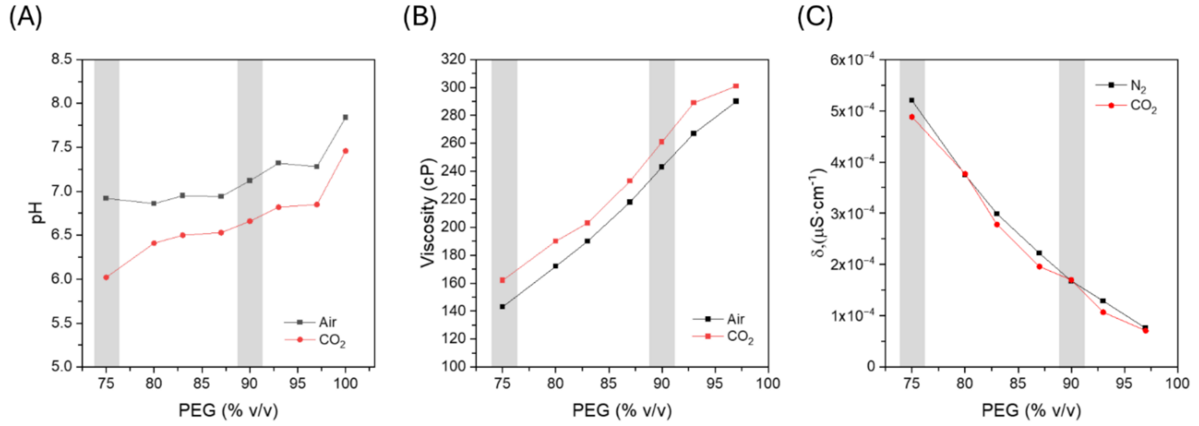

Figure S5. (A) pH, (B) Viscosity (C) Ionic conductivity of different compositions studied. Air atmosphere (black dot-line-dot); CO_2 atmosphere (dot-red line-dot).

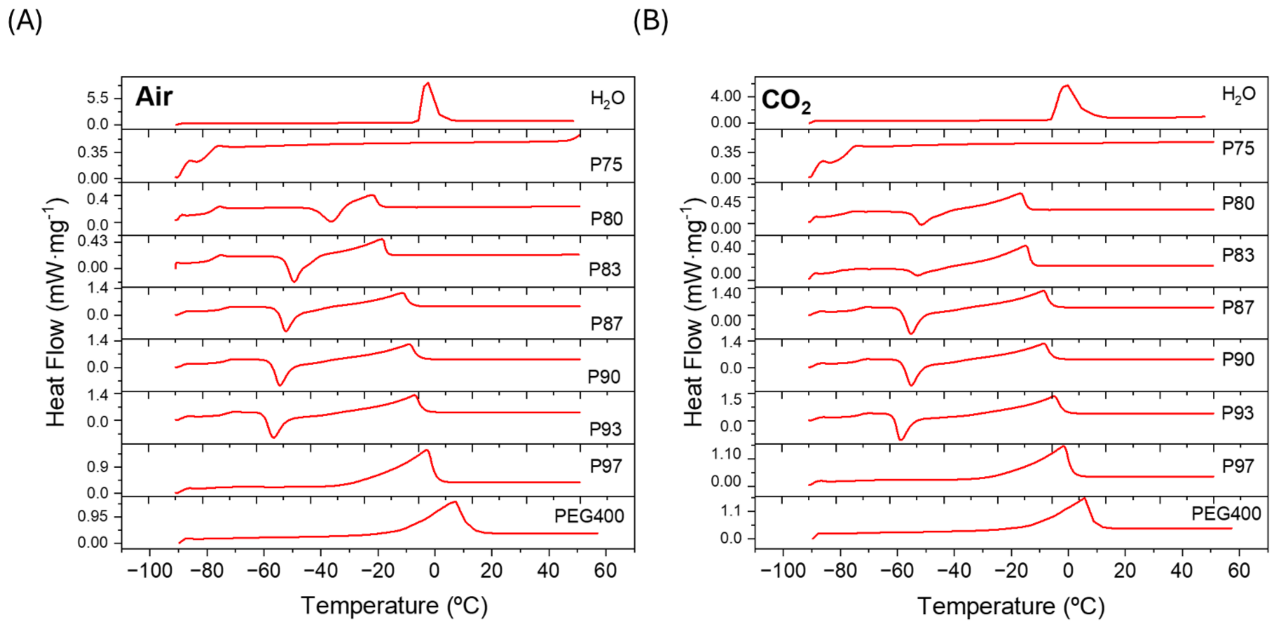

Figure S6. (A) DSC thermogram of PEG-H_2O-NaCl mixtures. (B) Air atmosphere; right, CO_2 atmosphere. 10ºC·min^{-1} from -90 ºC to 60 ºC.

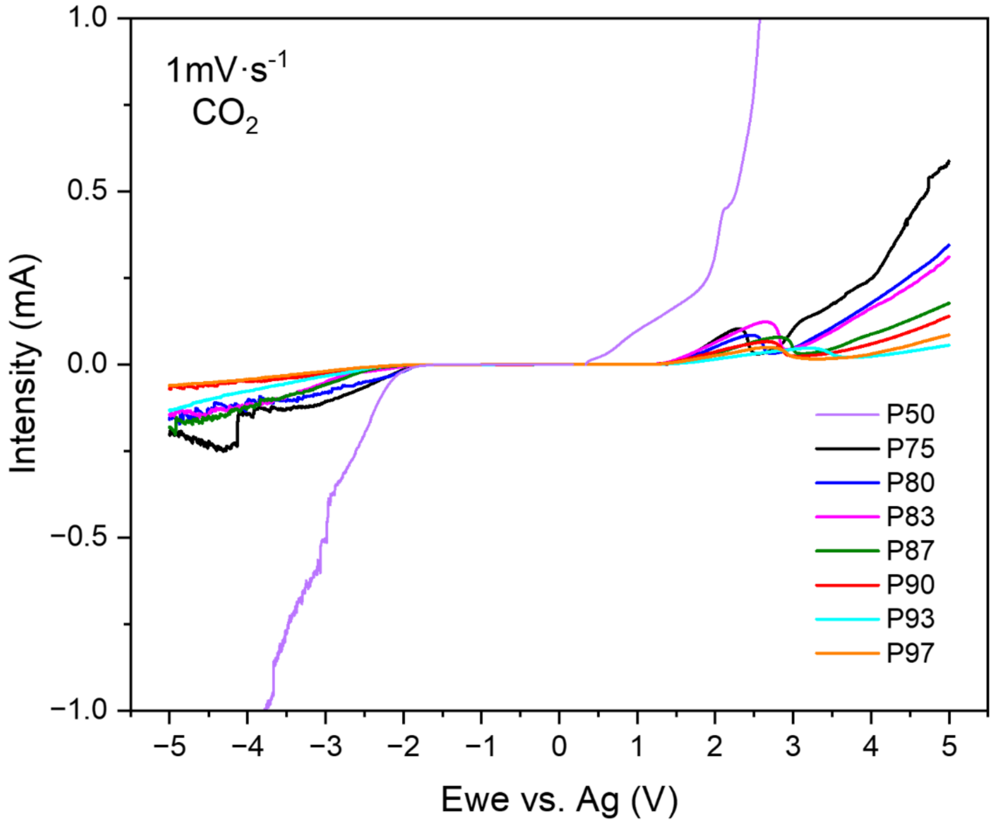

Figure S7. Electrochemical stability window in a three electrodes cell using LSV technique for PEG-H_2O-NaCl electrolytes for P50 composition with the PEG-H_2O-NaCl mixtures studied in CO_2 atmosphere. Scan rate: 1mV·s^{-1} Potential window studied: 5 V to -5 V vs. Ag.

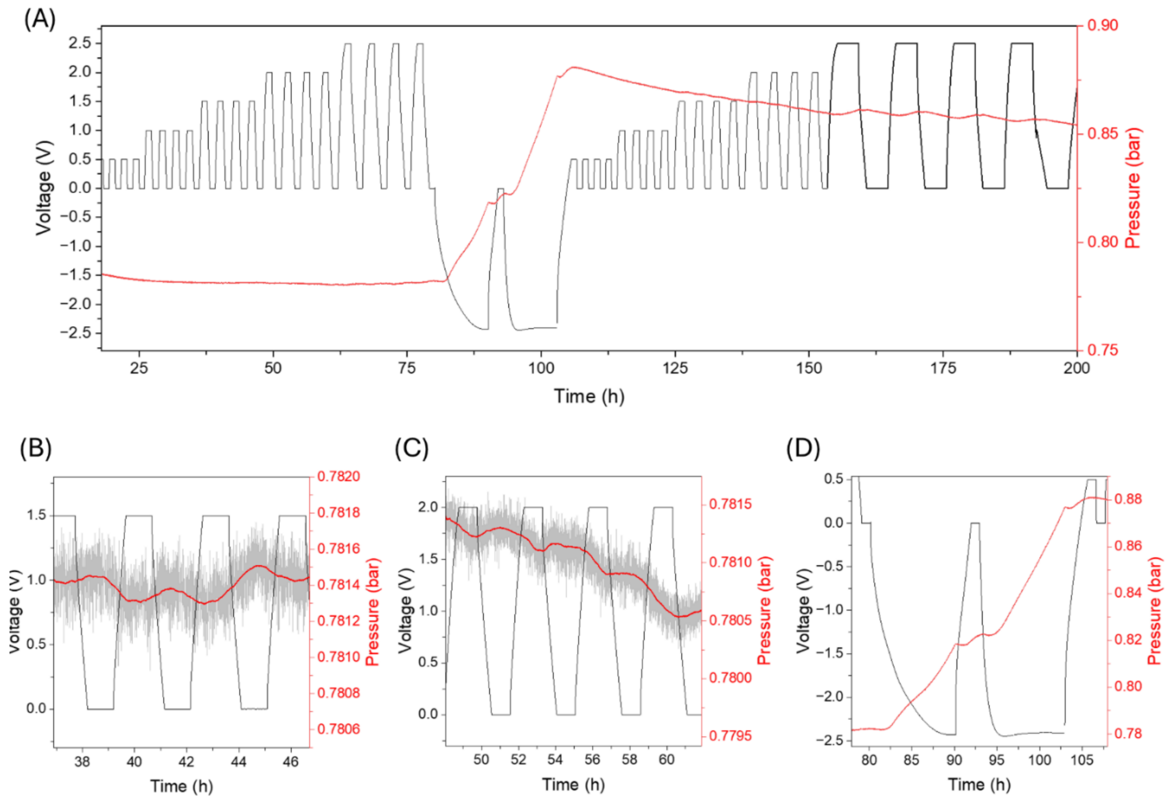

Figure S8. Electrochemical CO_2 capture measurement of P90 composition for voltage window study (A) Overall GCLP and pressure curves in a two-electrode cell at a current density value of 30 mA·g^{-1}, 1 and 4 hours of holding time, voltage windows: 0 – 0.5 V, 0 – 1.0 V, 0 – 1.5 V, 0 – 2.0 V, 0 - 2.5 V and -2.5 – 0 V. Zoomed GCLP and pressure curves (smoothed pressure curves for B and C) at voltage window (B) 0 to 1.5V (C) 0 to 2.0V (D) 0 to -2.5V. GCLP curves (black) and pressure curves (red).

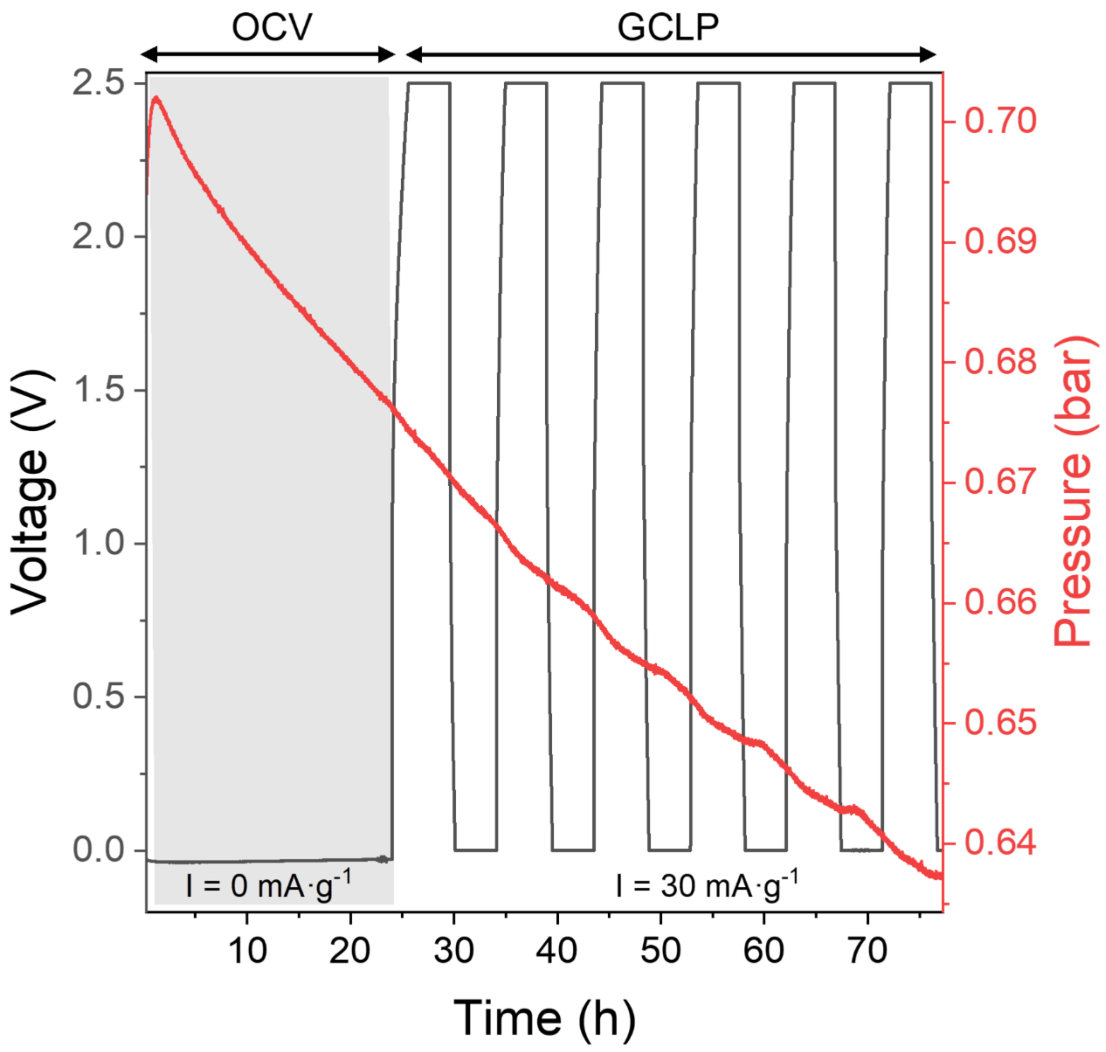

Figure S9. OCV and GCLP experiment to confirm that there is CO_2 capture only with the charging and discharging of the cell. 24 h of OCV measurement (shadow painted), and GCLP after OCV, 30 mA·g^{-1}; 4 h holding time; Voltage window 0 – 2.5 V; CO_2 atmosphere. Electrochemical curves (black) and pressure curves (red).

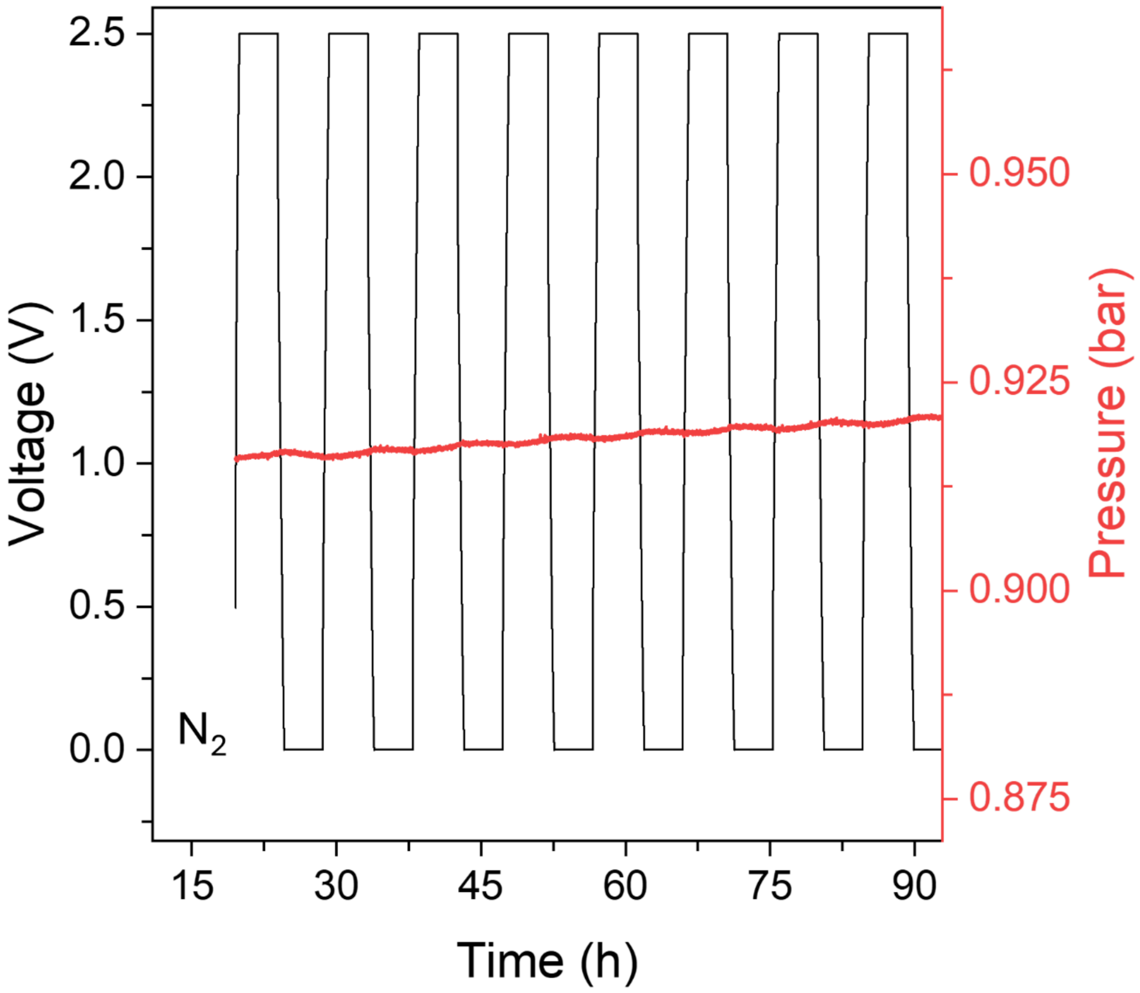

Figure S10. GCLP experiment with 2 electrodes cell; current density used, 30 mA·g^{-1}; 4 h holding time; Voltage window 0 - 2.5 V; N_2 atmosphere. GCLP curves (black) and pressure curves (red).

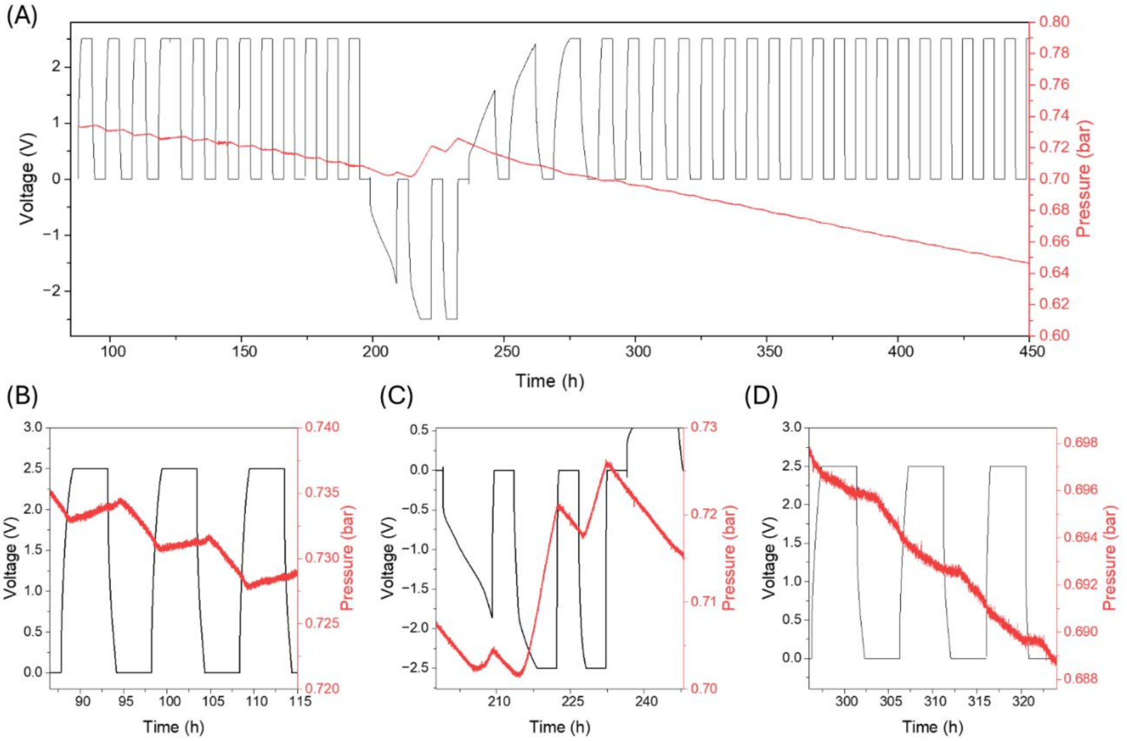

Figure S11. Electrochemical CO_2 capture measurement of **P75 composition** for current density study. (A) Overall galvanostatic cyclic limit potential (GCLP) curves at different current densities (30, 45, 60 and 75 mA·g^{-1}) and voltage window (0-2.5 V; 2.5-0 V). (B) Zoomed area corresponding to: (B) 30 mA·g^{-1}, voltage window from 0 to 2.5V in the first PCP; (C) 30 mA·g^{-1}, voltage window from -2.5V to 0 V in the NCP (D) 30 mA·g^{-1}, voltage window from 0 to 2.5 V in the second PCP. GCLP curves (black) and pressure curves (red).

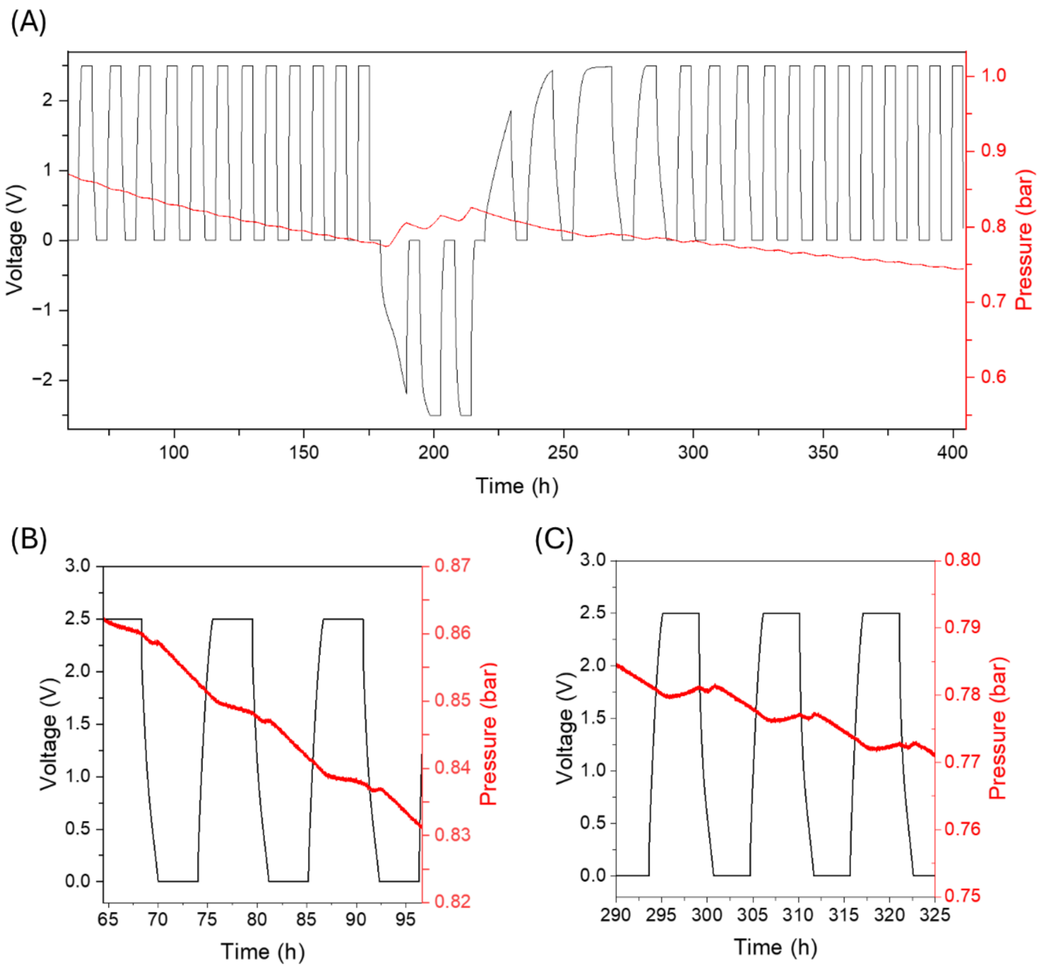

Figure S12. Electrochemical CO_2 capture measurement of **P90 composition** for current density study. (A) Overall galvanostatic cyclic limit potential (GCLP) curves at different current densities (30, 45, 60 and 75 mA·g^{-1}) and voltage window (0 – 2.5 V; -2.5 – 0 V). Zoomed area corresponding to: (B) 30 mA·g^{-1}, voltage window from 0 to 2.5 V in the first PCP;(C) 30 mA·g^{-1}, voltage window from 0 to 2.5V in the second PCP. GCLP curves (black) and pressure curves (red)

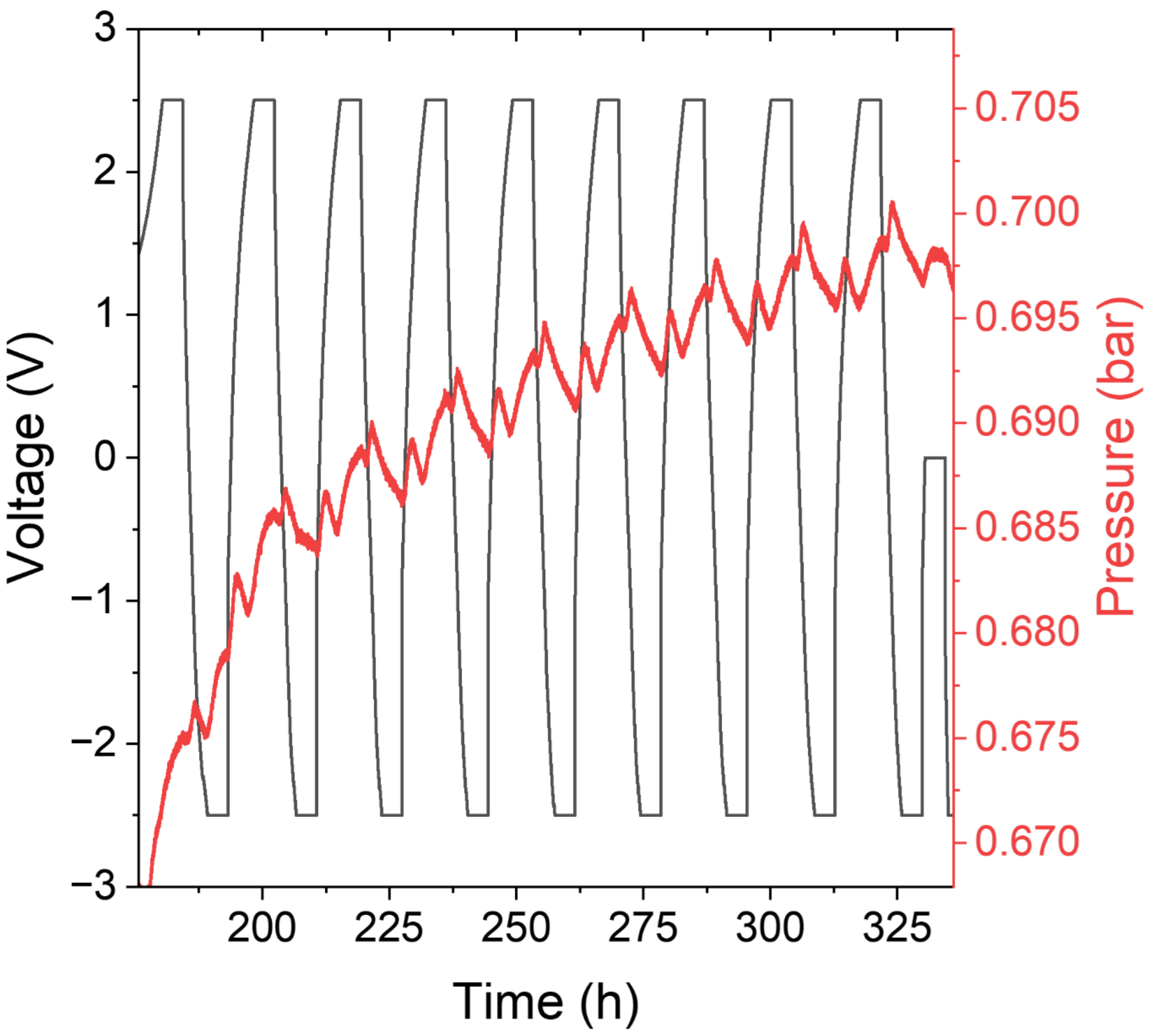

Figure S13. Electrochemical CO_2 capture measurement of **P90 composition** in Switch mode (Voltage window, from 2.5 to -2.5 V). Zoomed area corresponding to a GCLP and pressure curves in a two-electrode cell at a current density value of 30 mA·g^{-1}, 4 hours of holding time. GCLP curves (black) and pressure curves (red).

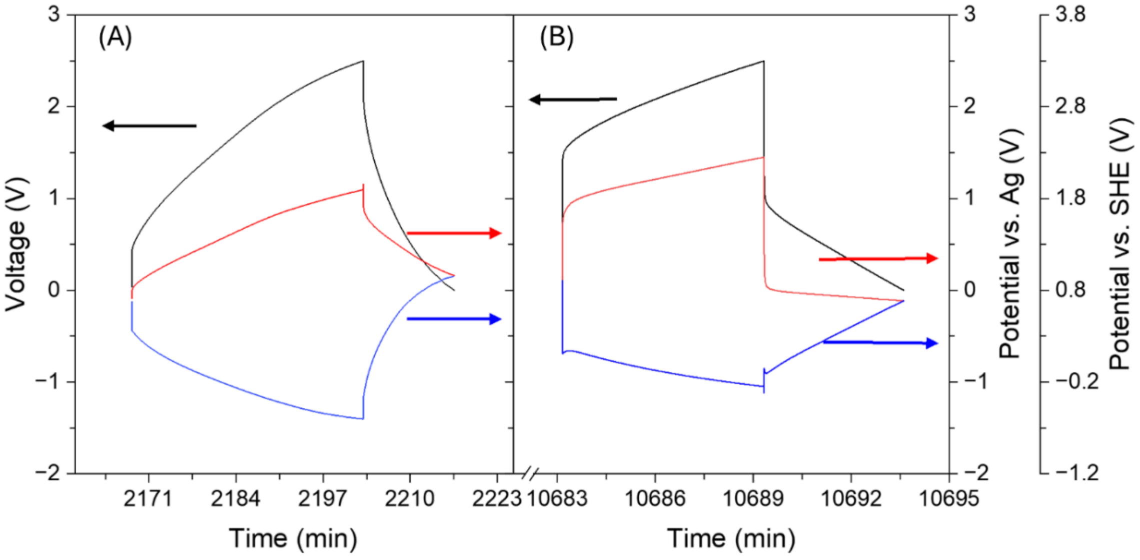

Figure S14. Three-electrode experiment of P75 symmetric supercapacitor. Potential variation of the cell (black), working electrode (red) and counter electrode (blue) in a GCLP measurement; current density, 30 $mA{\cdot}g^{-1}$; voltage window, 0 – 2.5 V. The potential variation of working electrode and counter electrodes are versus Ag bar. (A) Before change of polarization of the cell; (B) After change of polarization of the cell.

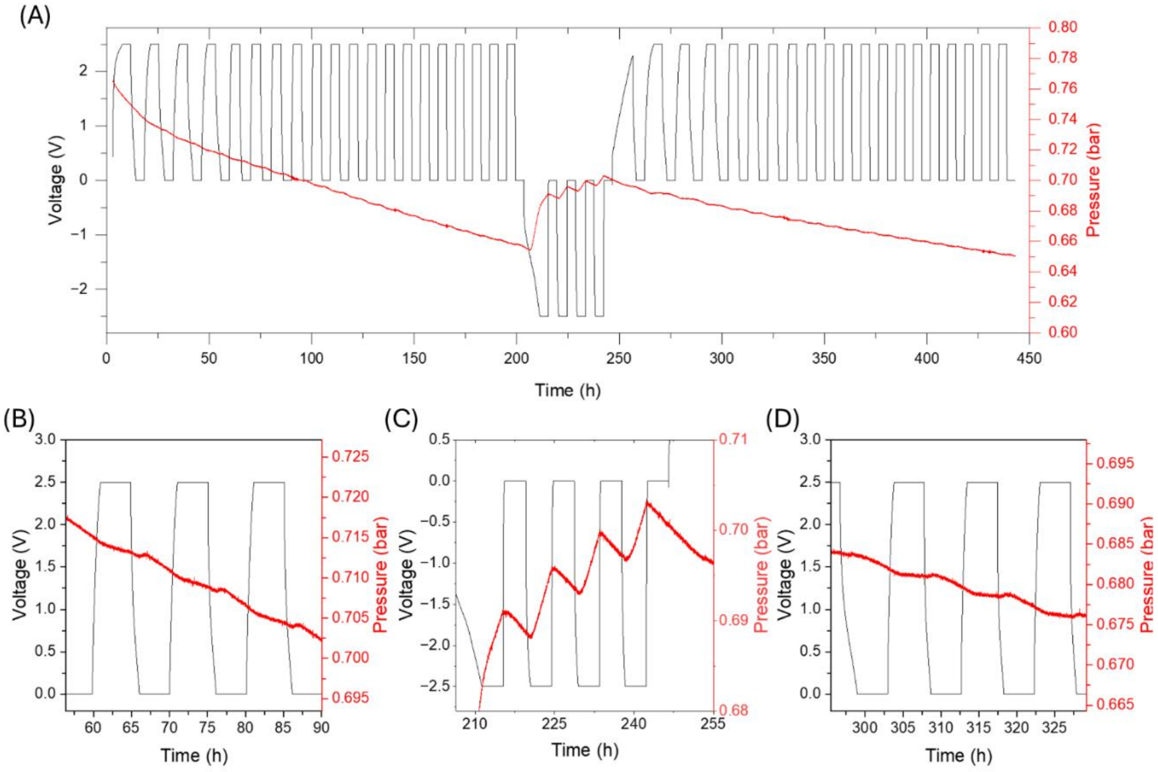

Figure S15. Electrochemical CO_2 capture measurement of P90MB (MB: mass balance) composition for potential window and holding time study in a two-electrode cell. (A) Overall galvanostatic cyclic limit potential (GCLP) curves at different current densities (30, 45, 60 and 75 $mA{\cdot}g^{-1}$) and voltage window (0 – 2.5 V; -2.5 – 0 V). Zoomed area corresponding to: (B) 30 $mA{\cdot}g^{-1}$, voltage window from 0 to 2.5V in the first PCP; (C) 30 $mA{\cdot}g^{-1}$, voltage window from 0 to -2.5 V in the NCP. (D) 30 $mA{\cdot}g^{-1}$, voltage window from 0 to -2.5 V in the NCP. GCLP curves (black) and pressure curves (red).

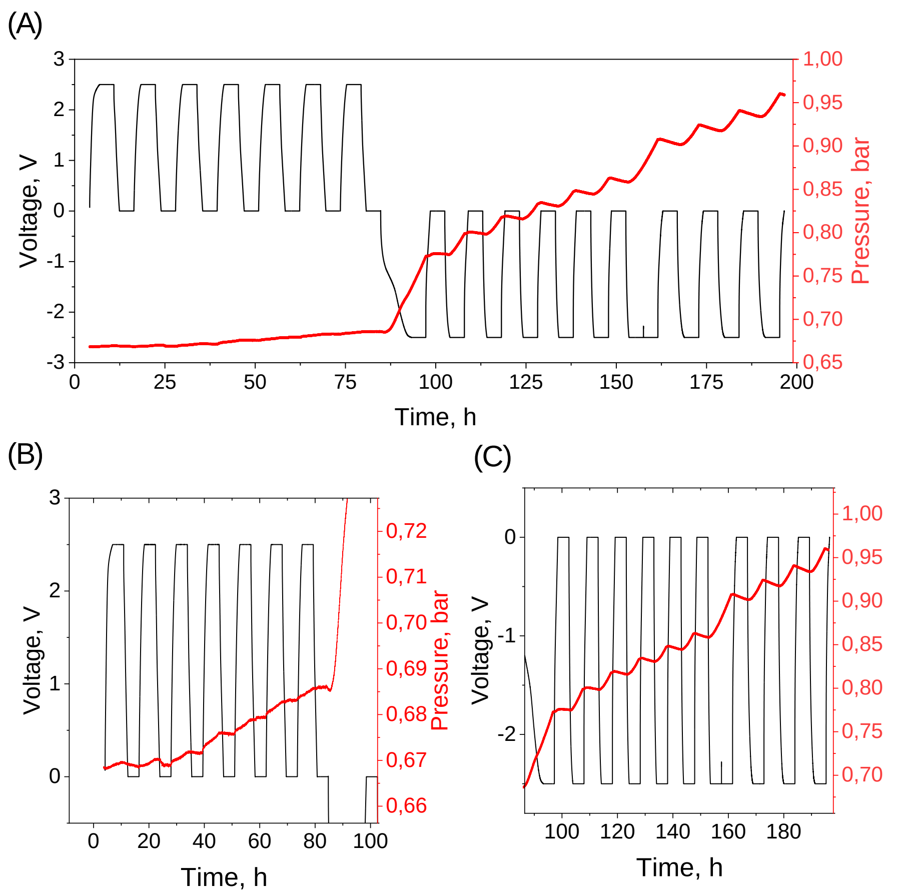

Figure S16. Electrochemical CO_2 capture measurement using P90 composition for a mixture of gases containing CO_2 (50%v/v) and air (50%v/v), with a potential window and holding time study in a two-electrode cell. (A) Overall galvanostatic cyclic limit potential (GCLP) curves at different current densities (30 and 45 mA·g^{-1}) and voltage window (0 – 2.5 V; -2.5 – 0 V). Zoomed area corresponding to: (B) 30 mA·g^{-1}, voltage window from 0 to 2.5V in the PCP; (C) 30 and 45 mA·g^{-1}, voltage window from 0 to -2.5 V in the NCP. GCLP curves (black) and pressure curves (red).

References.

[1] L. Wang, R. T. Yang, *Journal of Physical Chemistry C* 2012, *116*, 1099–1106.
